# Colloidal Phase Control in Plasmonic Metal Oxide Nanocrystals via Competitive Metal–Ligand Equilibria

**DOI:** 10.1002/anie.202518965

**Published:** 2025-11-04

**Authors:** Jiho Kang, Dingwen Qian, Jayoon Lee, Diana L. Conrad, Jessica D. Oberlander, M. Wren Berry, Jeffrey Liu, Eric V. Anslyn, Thomas M. Truskett, Delia J. Milliron

**Affiliations:** ^1^ McKetta Department of Chemical Engineering University of Texas at Austin Austin TX 78712 USA; ^2^ Department of Chemistry University of Texas at Austin Austin TX 78712 USA; ^3^ Department of Chemical Engineering University of Michigan Ann Arbor MI 48109 USA; ^4^ Biointerfaces Institute University of Michigan Ann Arbor MI 48109 USA

**Keywords:** Colloidal gel, Dynamic covalent bonding, Localized surface plasmon resonance, Metal coordination, Plasmon coupling

## Abstract

Colloidal nanocrystal gels offer tunable optical properties governed by both the nature of the building blocks and their spatial arrangement. When assembled via reversible molecular linkers, their phase behavior and structure are primarily dictated by bond strength and lability. However, precise control over these interactions remains a significant synthetic challenge and is often system‐specific. Here, we present a simple, broadly tunable linking strategy that modulates nanocrystal phase behavior and assembly structure by leveraging competitive metal–ligand equilibria. We achieve programmable control over gelation temperature and network structure by tuning competitive metal–terpyridine and metal–halide equilibria in terpyridine‐functionalized tin‐doped indium oxide (ITO) nanocrystals, governed by metal and halide identity, concentration, and temperature, enabling wide‐range infrared optical modulation. Combined kinetic Monte Carlo and optical simulations reveal that weaker, more labile links facilitate particle crawling, leading to denser gel structures with enhanced plasmon coupling. This strategy eliminates the need for complex ligand or linker design and establishes competitive coordination chemistry as a versatile platform for engineering dynamic, stimuli‐responsive colloidal assemblies.

## Graphical abstract

We achieve programmable gelation temperature and network structure in terpyridine‐functionalized tin‐doped indium oxide nanocrystals. This strategy leverages the tuning of competitive metal–terpyridine and metal–halide equilibria via metal and halide identity, concentration, and temperature, enabling broad‐range infrared optical modulation.

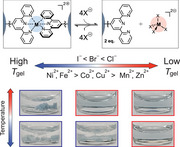

## Introduction

Colloidal nanocrystals, typically composed of inorganic cores and organic capping ligands, can exhibit size‐, shape‐, and composition‐dependent properties unattainable in bulk materials, such as the localized surface plasmon resonance (LSPR) of noble metal or degenerately doped metal oxide nanoparticles.^[^ ^1^, ^2^, ^3^ ^]^ Assembling nanocrystals enables structure‐dependent collective behavior that extends beyond individual particle properties.^[^ ^4^, ^5^, ^6^ ^]^ For example, gelation of tin‐doped indium oxide (ITO) nanocrystals leads to structure‐dependent plasmonic responses deviating from isolated particles, opening the door to switchable optical materials.^[^ ^7^, ^8^, ^9^, ^10^, ^11^, ^12^ ^]^ Theory and simulation predict that gel structure and the conditions for gelation and redispersion depend on a number of factors, including the strength and kinetics of crosslinking motifs.^[^ ^13^, ^14^, ^15^, ^16^ ^]^ However, a simple and modular experimental strategy to tune these parameters remains underdeveloped due to the synthetic complexity of the linking bonds.

Metal–ligand coordination is a form of dynamic covalent chemistry that offers facile synthetic tuning and bond reversibility, serving as a versatile linking strategy to construct macromolecules, polymer networks, and nanocrystal assemblies.^[^ ^17^, ^18^, ^19^, ^20^, ^21^, ^22^, ^23^ ^]^ Although dynamic covalent chemistry‐mediated molecular linking offers thermodynamic control over nanocrystal assembly through reversible, stimuli‐responsive bonds that can be tuned by linker‐to‐colloid ratio, binding strength, and linker length or rigidity,^[^ ^16^, ^24^, ^25^, ^26^, ^27^, ^28^, ^29^, ^30^, ^31^, ^32^, ^33^ ^]^ its application to nanocrystal assembly has been limited due to the need for customized synthesis of molecular libraries to vary these parameters. In contrast, metal–ligand coordination provides similar tunability through modular selection of linking components and their ratios. The Irving–Williams series describes the increasing stability of metal–ligand complexes across first‐row transition metals (${\mathrm{Mn}}^{2+}$ < ${\mathrm{Fe}}^{2+}$ < ${\mathrm{Co}}^{2+}$ < ${\mathrm{Ni}}^{2+}$ < ${\mathrm{Cu}}^{2+}$ > ${\mathrm{Zn}}^{2+}$),^[^ ^34^ ^]^ while the spectrochemical series ranks ligands by their field strength and denticity, which together influence complex stability (e.g., $I^{-}$ < ${\mathrm{Br}}^{-}$ < ${\mathrm{Cl}}^{-}$ < pyridine < bipyridine < terpyridine).^[^ ^35^ ^]^ This distinct metal complex stability has been exploited to create responsive materials. For example, reversible topological switching between linear and cross‐linked polymers has been achieved using ${\mathrm{Pd}}^{2+}$ as a metal linker, where ${\mathrm{Pd}}^{2+}$–triazole supramolecular bonds can be dissociated by introducing a competitive ligand, triphenylphosphine.^[^ ^36^ ^]^ Similarly, reversible assembly of gold nanoparticles functionalized with carboxylated peptides has been demonstrated using various metal ions (${\mathrm{Pb}}^{2+}$, ${\mathrm{Cd}}^{2+}$, ${\mathrm{Cu}}^{2+}$, and ${\mathrm{Zn}}^{2+}$) as linkers and a strong chelating agent, ethylenediaminetetraacetic acid, as a competitive ligand disrupting the interparticle linkages.^[^ ^37^ ^]^

Recently, we have demonstrated reversible gelation of ITO nanocrystals by a thermally and chemically responsive metal coordination linkage, achieving highly reproducible infrared optical modulation over thermal cycles.^[^ ^9^, ^11^, ^12^ ^]^ Gelation was driven by ${\mathrm{Co}}^{2+}$ coordinating with terpyridine (tpy) end groups of ligands anchored to the nanocrystal surface, while free‐flowing dispersions were recovered upon heating in the presence of sufficient chloride ions. This approach leverages a temperature‐dependent equilibrium shift between linking and non‐linking metal complexes, namely Co(tpy)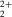 and ${\mathrm{CoCl}}_{4}^{2-}$, enabling the chemical modulation of gelation temperature (T_gel_) by varying the chloride ion concentration. However, a generalizable strategy to systematically tune the thermal stability of metal coordination linkages using different metal and halide ions has been lacking.

Herein, we extend this linking strategy based on competitive metal–ligand equilibria to diverse metal and halide ions, providing a modular platform to tune colloidal phase behavior and gel morphology. We use temperature‐controlled small‐angle X‐ray scattering (SAXS) to assess the thermal stability of nanocrystal gels across a broad parameter space, including chloride ion concentration, transition metal ion identity (${\mathrm{Mn}}^{2+}$, ${\mathrm{Fe}}^{2+}$, ${\mathrm{Co}}^{2+}$, ${\mathrm{Ni}}^{2+}$, ${\mathrm{Cu}}^{2+}$, and ${\mathrm{Zn}}^{2+}$) and halide ion identity ($I^{-}$, ${\mathrm{Br}}^{-}$, and ${\mathrm{Cl}}^{-}$). We find that the thermal stability of metal coordination linkages dictates both T_gel_ and gel morphology, enabling broad tunability of the infrared optical response. Moreover, in the presence of competitive ligands, metal‐terpyridine links become more labile, resulting in denser gels and enhanced LSPR coupling. Kinetic Monte Carlo simulations, combined with simulated optical response using a mutual polarization method (MPM),^[^ ^10^ ^]^ corroborate this trend, revealing that denser gels result from more dynamic linkages, as their short particle crawl times facilitate nanocrystal rearrangement. Finally, leveraging insights from competitive metal–ligand equilibria, we demonstrate reversible, stepwise tuning of T_gel_ and infrared optical states by sequential addition of different metal and halide ions.

## Results and Discussion

### Preparation and Assembly of Nanocrystal Building Blocks

**Figure 1:**
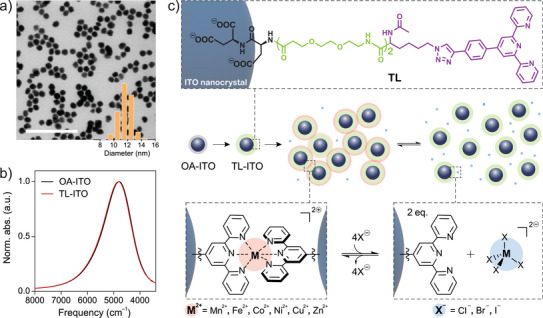
Preparation of TL‐ITO and nanocrystal assembly through metal coordination linkages. a) STEM image of ITO nanocrystals with their size distribution. Scale bar, 100 nm. b) LSPR absorption spectra of oleate‐capped ITO nanocrystals (OA‐ITO, in tetracholoroethylene) and ITO nanocrystals functionalized with terpyridine‐terminated ligands (TL‐ITO, in *N,N*‐dimethylformamide (DMF)). a.u., arbitrary units. c) Schematics showing functionalization of OA‐ITO with TL and assembly of TL‐ITO through metal‐coordination bonding using various transition metal ions (${\mathrm{Mn}}^{2+}$, ${\mathrm{Fe}}^{2+}$, ${\mathrm{Co}}^{2+}$, ${\mathrm{Ni}}^{2+}$, ${\mathrm{Cu}}^{2+}$, and ${\mathrm{Zn}}^{2+}$) and halide ions (${\mathrm{Cl}}^{-}$, ${\mathrm{Br}}^{-}$, and $I^{-}$). Here, nanocrystal assembly is controlled by the equilibrium between metal–(bis)terpyridine complexes and metal–tetrahalide complexes.

To prepare nanocrystal building blocks capable of metal coordination‐driven assembly, ITO nanocrystals were synthesized via a modified slow injection method and subsequently functionalized with terpyridine‐terminated ligands (TL) (Figure 1).^[^ ^8^, ^38^ ^]^ Bright‐field scanning transmission electron microscopy (BF‐STEM) images show monodisperse, quasi‐spherical ITO nanocrystals capped by native oleate ligands (OA‐ITO) (Figures 1a and S1). Size analyses from STEM images and SAXS of a dilute OA‐ITO dispersion in hexane, modeled with a spheroid form factor, revealed nanocrystal diameters of 11.7 ± 0.9 and 12.1 ± 1.2 nm, respectively (Figures 1a and S2). The Sn doping concentration, determined by inductively coupled plasma optical emission spectroscopy (ICP–OES), was found to be 4.8 at.% Sn. To enable nanocrystal assembly via metal‐coordination linkages, OA‐ITO were functionalized with TL, synthesized using established protocols to incorporate three functional domains: a tricarboxylate group serving as an anchor to the nanocrystal surface, a polyethylene oxide‐like chain for colloidal stability, and a terminal terpyridine group for metal coordination bonding (Figures 1c and S3).^[^ ^9^, ^11^, ^12^ ^]^ The Fourier transform infrared (FTIR) spectroscopy of TL‐ITO confirmed the presence of TL tethered to the nanocrystal surface via a tricarboxylate anchor, evidenced by characteristic terpyridine vibrational modes in the fingerprint region and the absence of carbonyl stretching bands associated with free carboxylic acid groups of unbound TL (Figure S4). The surface‐bound TL was further confirmed by comparing 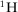 NMR spectra of TL and TL‐ITO in deuterated dimethyl sulfoxide (DMSO) (Figure S5). The characteristic terpyridine and amide peaks of TL between 7 and 9 parts per million (ppm) were substantially broadened in TL‐ITO,^[^ ^39^ ^]^ while a broad peak at 12.5 ppm, assigned to the carboxylic proton of unbound TL was absent, consistent with TL binding to the nanocrystal surface through its carboxylate group. The average number of bound TL per nanocrystal was estimated using 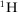 NMR on a mixed dispersion of TL‐ITO and an internal reference with known concentration (1,3,5‐trimethoxybenzene, 1 mM) (Figure S5). Quantification by comparing integrated peak areas of 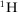 NMR yielded 190 bound TL per nanocrystal, although this value should be considered an approximation due to potential H/D exchange of the amide proton with residual water, even in non‐protic solvents such as deuterated DMSO. Thermogravimetric analysis of TL‐ITO revealed that organic ligands (oleate and TL combined) accounted for 13.6 wt.% of the nanocrystal mass, yielding a ligand molar ratio of 8:1 (oleate:TL) (Figure S6). Infrared absorption spectra and SAXS patterns of ITO nanocrystal dispersions before and after TL functionalization remained nearly unchanged, indicating that their size and plasmonic properties were not affected by ligand exchange (Figures 1b and S2). Adding anhydrous metal dichloride salts (${\mathrm{MCl}}_{2}$, M = Mn, Fe, Co, Ni, Cu, or Zn) to a dispersion of TL‐ITO facilitates the formation of metal(II)‐(bis)terpyridine (M(tpy)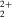) linkages, leading to nanocrystal gelation (Figure 1c). However, when excess halide ions such as ${\mathrm{Cl}}^{-}$, ${\mathrm{Br}}^{-}$, or $I^{-}$ are present, a competitive equilibrium is established between the linking M(tpy)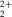 complexes and non‐linking metal‐tetrahalide species (${\mathrm{MCl}}_{4}^{2-}$). In our gelation strategy, this competitive equilibrium governs the prevalence of effective linkages between nanocrystals and dictates whether particles remain dispersed or undergo assembly.^[^ ^9^ ^]^

### Phase Control by Metal Ion Identity and Chloride Concentration

**Figure 2:**
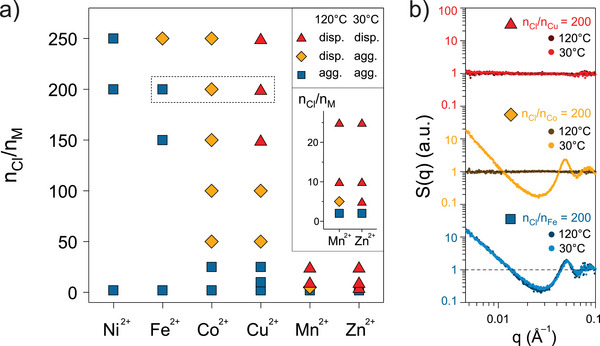
Phase behavior of TL‐ITO with various transition metal ions. a) Phase behavior of TL‐ITO as a function of the chloride‐to‐metal ion molar ratio ($n_{Cl}/n_{M}$), with an inset showing a zoomed‐in view. Blue squares represent nanocrystals that form gels at both 30 and 120 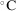. Yellow diamonds indicate nanocrystals that form gels at 30 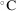 but are dispersed at 120 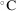. Red triangles denote nanocrystals that remain dispersed at both 30 and 120 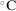. The concentration of metal dichloride salt (${\mathrm{MCl}}_{2}$, M = Mn, Fe, Co, Ni, Cu, and Zn) in each sample was maintained at 2 mM, while the chloride concentration was varied by adding different amounts of tetrabutylammonium chloride (TBACl). The molar concentration of TL‐ITO and surface‐bound TL was fixed at 1.7 and 320 μM, respectively. b) SAXS structure factors S(q) for samples in the dashed box in (a) at 30 and 120 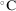, offset for clarity. Horizontal dashed lines indicate S(q) = 1.

The thermal stability of nanocrystal gels at different chloride concentrations varied markedly with metal ion identity, reflecting metal‐dependent tuning of the abundance of linking and non‐linking metal complexes. To investigate how metal ion identity influences thermal stability of gels, the colloidal phase of TL‐ITO was monitored by SAXS at two thermal setpoints (30 and 120 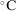) after adding various metal dichloride salts and varying chloride‐to‐metal ratio ($n_{Cl}/n_{M}$) (Figures 2 and S7–S9). Deviations of SAXS structure factor, S(q), from 1 reveal the average organization of the nanocrystal assembly across varying interparticle distances, where q is inversely proportional to the length scale. Based on whether nanocrystals exhibited diverging SAXS S(q) at low q at each temperature (low–high), their colloidal phase behavior was categorized into three response types: assembled–assembled (AA), assembled–dispersed (AD), and dispersed–dispersed (DD) (Figure 2b). Our previous work with ${\mathrm{Co}}^{2+}$ as the metal linker spectroscopically showed that increasing temperature or chloride ion concentration gradually shifts the equilibrium from Co(tpy)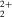 to ${\mathrm{CoCl}}_{4}^{2-}$.^[^ ^9^ ^]^ Consistent with this thermodynamic framework, all TL‐ITO dispersions, regardless of the metal ion used, exhibited a unidirectional transition from AA to AD to DD with increasing $n_{Cl}/n_{M}$ (Figure 2a). However, the specific $n_{Cl}/n_{M}$ required to induce these transitions varied markedly with metal ion identity. For example, ${\mathrm{Ni}}^{2+}$‐linked samples persisted in the AA state even at $n_{Cl}/n_{M}$ = 250, indicating remarkable thermal stability due to a strong preference for forming linking Ni(tpy)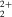 complexes over non‐linking ${\mathrm{NiCl}}_{4}^{2-}$. In contrast, the AD state was observed for samples with ${\mathrm{Fe}}^{2+}$, ${\mathrm{Co}}^{2+}$, ${\mathrm{Cu}}^{2+}$, and ${\mathrm{Mn}}^{2+}$ linkers at progressively lower $n_{Cl}/n_{M}$ values (250, 50, 50, and 5, respectively). The diminishing $n_{Cl}/n_{M}$ threshold for the AD state along the series suggests that particles are more likely to exist in free‐flowing dispersion states at elevated temperature due to increasing tendency to form non‐linking ${\mathrm{MCl}}_{4}^{2-}$ complexes. The onset of DD state followed a similar trend, occurring at $n_{Cl}/n_{M}$ > 150 for ${\mathrm{Cu}}^{2+}$, 10 for ${\mathrm{Mn}}^{2+}$, and 5 for ${\mathrm{Zn}}^{2+}$. Notably, ${\mathrm{Zn}}^{2+}$‐containing samples showed an abrupt transition directly from AA to DD between $n_{Cl}/n_{M}$ = 2 and 5, suggesting a particularly strong preference for forming non‐linking ${\mathrm{ZnCl}}_{4}^{2-}$ complexes over Zn(tpy)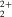. Altogether, the tendency to favor linking metal–terpyridine complexes over non‐linking metal–tetrahalide complexes increased along the series ${\mathrm{Ni}}^{2+}$ > ${\mathrm{Fe}}^{2+}$ > ${\mathrm{Co}}^{2+}$ > ${\mathrm{Cu}}^{2+}$ > ${\mathrm{Mn}}^{2+}$ ≥ ${\mathrm{Zn}}^{2+}$, establishing metal ion type as a crucial control parameter for the competitive metal–ligand equilibria beyond chloride concentration and temperature. This trend also generally aligns with the overall stability constants of transition metal ion–bis(terpyridine) complexes in water: ${\mathrm{Ni}}^{2+}$ > ${\mathrm{Fe}}^{2+}$ > ${\mathrm{Co}}^{2+}$ > ${\mathrm{Zn}}^{2+}$.^[^ ^40^, ^41^ ^]^

### Gel Structure and Infrared Optical Modulation

**Figure 3:**
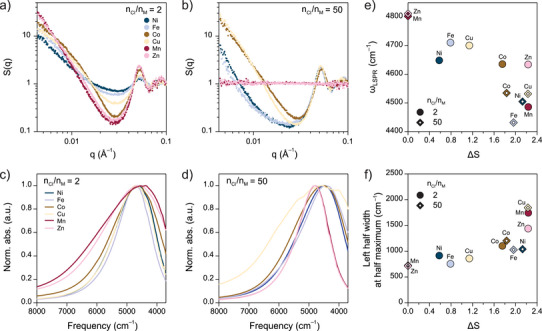
Structural and infrared optical modulation of TL‐ITO through metal‐coordination tuning. a,b) SAXS S(q) of nanocrystal assemblies using various divalent metal ions at chloride‐to‐metal ratio ($n_{Cl}/n_{M}$) of (a) 2 and (b) 50. c,d) LSPR absorption spectra of nanocrystal assemblies using various divalent metal ions at $n_{Cl}/n_{M}$ of (c) 2 and (d) 50. The fine structures near the absorption peaks are attributed to residual solvent background signals. e,f) LSPR peak frequency $\omega_{\text{LSPR}}$ (e) and left half width at half maximum (f) of nanocrystal assemblies as a function of $\Delta S=S\left(q_{1}\right)-S\left(q_{2}\right)$, where $q_{1}$ and $q_{2}$ are q of primary peak and the minimum of porod dip in S(q), respectively. The concentration of TL‐ITO and metal dichloride salt (${\mathrm{MCl}}_{2}$, M = Mn, Fe, Co, Ni, Cu, and Zn) in each sample was maintained at 1.7 μM and 2 mM, respectively.

Beyond simple phase control between dispersed and gel states, our linking strategy leveraging competitive metal–ligand equilibrium facilitates broad, continuous modulation of gel structures and infrared optical responses through tunable bond lability. To investigate this, SAXS patterns and LSPR absorption spectra were collected from TL‐ITO samples with different metal ions at two distinct chloride concentrations ($n_{Cl}/n_{M}$ = 2 and 50) (Figure 3). Although all samples formed nanocrystal assemblies at $n_{Cl}/n_{M}$ = 2, their structures varied significantly with metal ion identity (Figure 3a). For instance, ${\mathrm{Mn}}^{2+}$‐ and ${\mathrm{Zn}}^{2+}$‐linked gels, which exhibited low thermal stability (Figure 2a), yielded the most coarsened gel structures, as indicated by the largest difference between the primary peak and the minimum of the Porod dip (ΔS) in SAXS S(q), a parameter that describes the magnitude of spatial correlations between neighboring particles.^[^ ^42^ ^]^ In contrast, thermally stable assemblies with ${\mathrm{Ni}}^{2+}$ and ${\mathrm{Fe}}^{2+}$ linkers formed the least coarsened gels, while ${\mathrm{Co}}^{2+}$‐ and ${\mathrm{Cu}}^{2+}$‐linked gels with intermediate thermal stability showed moderately coarsened structures. Given that all samples remained in the AA state at $n_{Cl}/n_{M}$ = 2 irrespective of metal ion type (Figure 2a), it is evident that the linking metal–terpyridine complexes dominate the competitive equilibrium at such low chloride concentrations and ambient temperature, resulting in gelation through gas–liquid spinodal decomposition. The arrested gel structure is under kinetic control, yielding a more coarsened structure when the system is quenched less deeply from the gelation boundary in the phase diagram, i.e., for relatively weakly bound metal–terpyridine complexes.^[^ ^43^ ^]^ For colloidal nanocrystal gels with strong short‐range attractions, this structural coarsening is typically driven by nanocrystal crawling, the slow rearrangement of linked particles around each other without requiring particles to dissociate from and reattach to the network.^[^ ^44^ ^]^

Increasing chloride ion concentration lowers gelation temperature by shifting the competitive equilibrium toward non‐linking complexes, effectively rendering the system less deeply quenched from the gelation boundary in the phase diagram and facilitating structural coarsening (Figures 2a and 3b). Consequently, gels with ${\mathrm{Ni}}^{2+}$, ${\mathrm{Fe}}^{2+}$, ${\mathrm{Co}}^{2+}$, and ${\mathrm{Cu}}^{2+}$ linkers at $n_{Cl}/n_{M}$ = 50 exhibited denser structures than their counterparts at $n_{Cl}/n_{M}$ = 2 (Figure 3b). Notably, ${\mathrm{Ni}}^{2+}$‐ and ${\mathrm{Fe}}^{2+}$‐linked gels, which resulted in higher thermal stability compared to ${\mathrm{Co}}^{2+}$‐ and ${\mathrm{Cu}}^{2+}$‐linked gels, exhibited superlinear S(q) at low q at $n_{Cl}/n_{M}$ = 50, indicative of heterogeneous gel structures with pronounced mesoscale density fluctuations. Meanwhile, TL‐ITO with ${\mathrm{Mn}}^{2+}$ and ${\mathrm{Zn}}^{2+}$ linkers remained fully dispersed at $n_{Cl}/n_{M}$ = 50, indicating that the systems are positioned above the gelation boundary as non‐linking metal–halide complexes outcompete linking metal–terpyridine complexes (Figures 2a and 3b).

The infrared optical response of TL‐ITO nanocrystal gels is directly correlated with and broadly tunable by their mesoscale structural coarsening, which can be precisely controlled using chemical parameters. Importantly, all assembled samples, regardless of metal ion or chloride concentration, exhibited similar characteristic lengths for nearest neighbor spacing, reflected in the position of the primary peak in S(q), indicating that the variation in collective plasmonic response arises primarily from mesoscale structural coarsening rather than differences in interparticle spacing (Figure S10).^[^ ^12^ ^]^ At $n_{Cl}/n_{M}=2$, LSPR absorption spectra were increasingly redshifted and broadened with weaker metal ion linkers, consistent with enhanced plasmon coupling in denser gel structures (Figures 3c and S11). Likewise, at $n_{Cl}/n_{M}=50$, gels with intermediate and strong linkers exhibited more pronounced features of LSPR coupling compared to their low‐chloride counterparts (Figures 3d and S12). The fully dispersed TL‐ITO with weak‐binding ${\mathrm{Mn}}^{2+}$ and ${\mathrm{Zn}}^{2+}$ linkers displayed infrared absorption spectra identical to TL‐ITO dispersions without linkers (Figures 1b and 3d). Monitoring the spectral peak features as a function of ΔS provided a more evident view of spectral modulation achieved by controlling the colloidal phase and gel structure (Figure 3e,f). Regardless of the metal ion identity or chloride concentration, the peak position ($\omega_{\text{LSPR}}$) redshifted with increasing ΔS, enabling continuous tuning of $\omega_{\text{LSPR}}$ from above 4800 ${\mathrm{cm}}^{-1}$ to below 4500 ${\mathrm{cm}}^{-1}$ (Figure 3e). A similar trend was observed for left half width at half maximum as a function of ΔS, demonstrating a continuous tunability in spectral width of more than two‐fold, spanning from dispersed states to coarsened gels (Figure 3f). Such control over both spectral position and width offers an effective strategy to tailor infrared optical response of nanocrystal gels using simple, modular chemical inputs.

### Effect of Bond Lability on Gel Structure and Optical Properties

**Figure 4:**
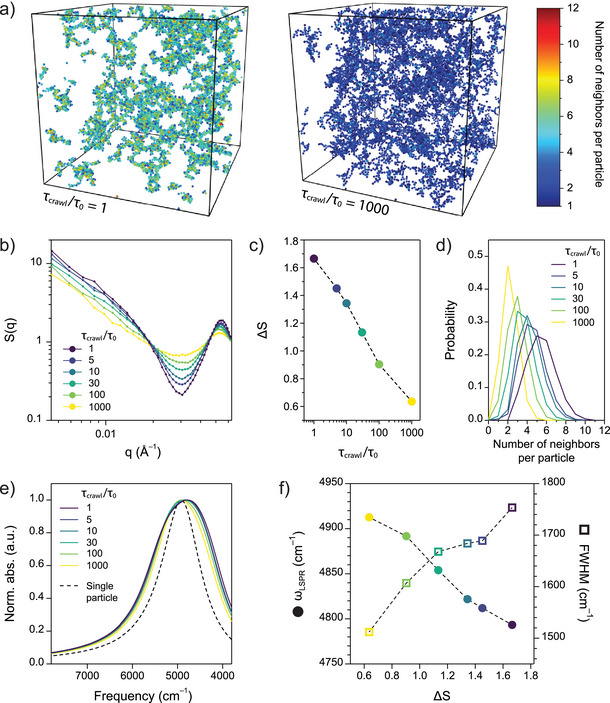
Local gel structure and optical evolution as a function of particle crawling time a) Snapshots from kinetic Monte Carlo (kMC) simulations showing nanocrystal assembly with particle crawling times $\tau_{\text{crawl}}=\tau_{0}$ (left) and $\tau_{\text{crawl}}=1000\tau_{0}$ (right). The depth of the interaction potential is fixed at ε=−20kT, reflecting the presence of strong short‐range interactions. The color scale indicates the number of neighbors per particle. b) Static SAXS structure factor S(q) calculated from kMC simulation at the onset of percolated clusters. c) Difference between the primary peak and the Porod dip in S(q), denoted as ΔS, plotted as a function of $\tau_{\text{crawl}}$. d) The number of neighbors per particle with different $\tau_{\text{crawl}}$. e) LSPR extinction spectra of percolated structures calculated using the mutual polarization method. Single particle spectrum (dashed) is shown for reference. f) LSPR peak frequency ($\omega_{\text{LSPR}}$, filled circles) and full width at half maximum (FWHM, empty squares) of simulated extinction spectra as a function of ΔS.

Kinetic Monte Carlo (kMC) and optical simulations revealed how bond lability critically controls local gel coarsening, directly influencing the infrared optical properties of the assembled nanocrystal networks. We first focus on the low chloride concentration condition ($n_{Cl}/n_{M}$ = 2, Figure 3a), where crawling is hypothesized to be the main mechanism of coarsening. As the metal–terpyridine complexes become more stable, the kinetics of crawling are expected to slow, leading to an increase in the characteristic crawling time relative to the diffusion timescale $\tau_{\text{crawl}}/\tau_{0}$ (Supporting Information S4.1). To better understand the influence of crawling kinetics on the coarsening process, kMC simulations were conducted across a range of particle crawling times (Figure 4). Simulated percolated networks at smaller $\tau_{\text{crawl}}/\tau_{0}$ exhibited denser structures with increased number of neighbors per particle (Figure 4a,d). Notably, the average number of neighbors per particle increased from approximately 2 to 6 as $\tau_{\text{crawl}}/\tau_{0}$ decreased from 1000 to 1, indicating a structural transition of the nanocrystal network from a fractal to a denser, bicontinuous morphology. Simulated static SAXS S(q) further corroborated this trend, showing that ΔS is monotonically increasing with smaller $\tau_{\text{crawl}}/\tau_{0}$ (Figure 4b,c). The concurrent increase of ΔS with the average number of neighbors per particle reinforces its validity as a descriptor of spatial correlations at the nearest‐neighbor length scale. These results indicate that bond lability, which can be tuned by changing the metal ion identity, serves as a key parameter for controlling local gel coarsening through its influence on crawling kinetics (Figure 3a). To evaluate the impact of local gel coarsening on the LSPR spectra, optical simulations were performed using MPM for percolated networks with varying $\tau_{\text{crawl}}/\tau_{0}$ (Figure 4e). Although all gel structures exhibited spectral redshift and broadening relative to the single‐particle spectrum, stronger plasmon coupling emerged in networks with smaller $\tau_{\text{crawl}}/\tau_{0}$ as higher coordination number leads to greater plasmonic coupling. Consistent with experimental observation, peak position ($\omega_{\text{LSPR}}$) and full width at half maximum (FWHM) of simulated spectra decreased and increased, respectively, with increasing ΔS (Figure 4f). This monotonic trend establishes strong connections between the structural and optical characteristics of the systems. For the high chloride concentration condition ($n_{Cl}/n_{M}$ = 50, Figure 3b), it is more probable for the nanocrystals to dissociate and reattach due to the shift of the competitive equilibrium toward non‐linking complexes. The dissociation and reattachment of nanocrystals—referred to as hopping in the literature—may facilitate more efficient coarsening during the gelation process.^[^ ^44^ ^]^ We hypothesize that enhanced hopping at elevated chloride concentrations contributes to the formation of denser structures observed experimentally relative to their lower chloride counterparts (Figure 3a,b). This hypothesis is supported by simulation results, which show that nanoparticles assemble into denser networks at lower attraction strengths, where hopping events occur more frequently during gelation (Figure S13).

### Phase Control by Halide Ion Identity

**Figure 5:**
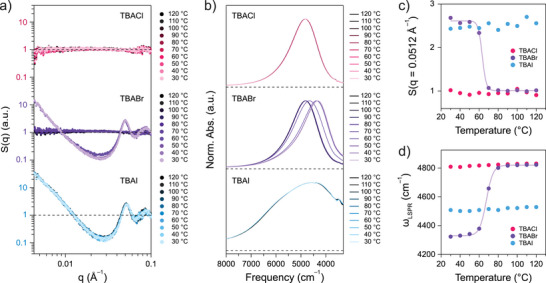
Control of nanocrystal phase behavior using different halide ions. a) SAXS S(q) and b) infrared response of 1.7 μM TL‐ITO with 2 mM ${\mathrm{ZnCl}}_{2}$ and 10 mM tetrabutylammonium salts with different halide ions: TBACl (top), TBABr (middle), and TBAI (bottom), offset for clarity. Horizontal dashed lines in (a) and (b) indicate S(q) = 1 and absorbance = 0, respectively. c) The intensity of the primary SAXS S(q) peak and d) LSPR peak frequency ($\omega_{\text{LSPR}}$) of the corresponding nanocrystal assemblies. Sigmoid fits are used to determine T_gel_ of samples containing TBABr in (c) and (d), yielding values of 63.3 and 67.5 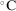, respectively.

Besides its concentration, the identity of competitive ligands substantially influenced the equilibrium between linking and non‐linking metal complexes, leading to distinct colloidal phase behavior. We hypothesized that halides with weaker ligand strength would be less likely to form non‐linking metal complexes, thus facilitating nanocrystal assembly via M(tpy)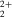 linkages. To test this, SAXS patterns and infrared response were collected from TL‐ITO samples after adding ${\mathrm{Zn}}^{2+}$ along with ${\mathrm{Cl}}^{-}$, ${\mathrm{Br}}^{-}$, or $I^{-}$ under otherwise identical conditions (Figure 5). Indeed, nanocrystal assemblies exhibited higher thermal stability as halide ligand strength decreased. The SAXS S(q) of the ${\mathrm{Cl}}^{-}$‐containing sample remained near unity at all temperatures, indicating a fully dispersed state due to preferred formation of ${\mathrm{ZnCl}}_{4}^{2-}$. In contrast, nanocrystals with the weakest halide, $I^{-}$, maintained unchanging fractal gel structures at all temperatures, as evidenced by the sustained divergence of S(q) at low q in the semi‐log plot, implying that the ligand strength of iodide is insufficient to disrupt zinc‐terpyridine linkages. Whereas the colloidal state of TL‐ITO with ${\mathrm{Cl}}^{-}$ or $I^{-}$ remained the same across temperatures due to strong preference for either non‐linking or linking zinc complexes, ${\mathrm{Br}}^{-}$, a halide with intermediate ligand strength, enabled a temperature‐dependent transition in the dominant zinc species. The ${\mathrm{Br}}^{-}$‐containing sample exhibited near‐unity S(q) above 70 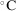, but underwent abrupt gelation at 60 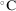. This abrupt phase transition, in contrast to the gradual increase in linking zinc complexes with decreasing temperature, aligns with our previous finding that gelation occurs once the number of effective links exceeds a critical threshold.^[^ ^9^ ^]^

Temperature‐dependent colloidal state was also evident in the infrared absorption spectra (Figure 5b). The ${\mathrm{Cl}}^{-}$‐containing sample maintained an LSPR profile resembling that of isolated nanocrystals across all temperatures, indicating negligible particle aggregation. In contrast, the $I^{-}$‐containing sample exhibited redshifted and broadened infrared absorption spectra at all temperatures, characteristic of collective plasmon resonance in assembled ITO nanocrystals. Consistent with the phase transition observed in SAXS, a ${\mathrm{Br}}^{-}$‐containing sample showed spectral evolution with decreasing temperature, with LSPR peak frequency ($\omega_{\text{LSPR}}$) shifting from 4800 ${\mathrm{cm}}^{-1}$ to below 4400 ${\mathrm{cm}}^{-1}$. T_gel_ was determined by the inflection point of a sigmoid fit for both S(q) primary peak intensity and $\omega_{\text{LSPR}}$, yielding closely matched values of 63.3 and 67.5 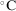, respectively (Figure 5c,d).

### Sequentially Reprogrammable Nanocrystal Phase Behavior

**Figure 6:**
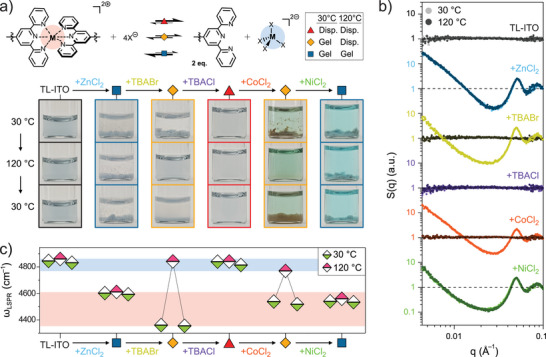
Continuous tuning of nanocrystal phase behavior. a) Schematics illustrating the equilibrium between metal–(bis)terpyridine and metal–tetrahalide complexes (top), along with digital photos of a TL‐ITO dispersion in DMF at various stages of sequential additive introduction: ${\mathrm{ZnCl}}_{2}$, TBABr, TBACl, ${\mathrm{CoCl}}_{2}$, and ${\mathrm{NiCl}}_{2}$ (bottom). Photos were taken at 30 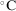 initially, at 120 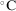 after heating, and again at 30 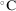 after cooling. b) SAXS S(q), offset for clarity, and c) $\omega_{\text{LSPR}}$ of TL‐ITO dispersions corresponding to each step of the cumulative addition process during the thermal cycle. Horizontal dashed lines in (b) indicate S(q) = 1. The SAXS patterns and infrared optical response were collected using six individually prepared samples to avoid influencing the analysis with the dilution effects that occur during the sequential chemical additions, maintaining the concentrations of TL‐ITO and dichloride salts at 1.7 μM and 2 mM, respectively.

Leveraging our understanding of the competitive metal–ligand equilibria, we aimed to design a system where T_gel_ can be reversibly modulated by the sequential addition of metal and halide ions (Figure 6). We reasoned that successive additions of stronger metal or halide ions would override the effect of previously established equilibria, enabling reversible modulation of nanocrystals' phase behavior. To test this, the thermal stability of TL‐ITO was monitored through visual inspection after sequentially adding ${\mathrm{ZnCl}}_{2}$, TBABr, TBACl, ${\mathrm{CoCl}}_{2}$, and finally ${\mathrm{NiCl}}_{2}$ (Figure 6a). Without a metal linker, nanocrystals remained colloidally stable at both 30 and 120 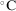. Upon addition of ${\mathrm{ZnCl}}_{2}$, however, nanocrystals instantly aggregated into a gel that maintained its structure during thermal cycling, as confirmed by unchanging gel features such as a diverging SAXS S(q) at low q and a redshifted $\omega_{\text{LSPR}}$ relative to TL‐ITO dispersion by over 200 ${\mathrm{cm}}^{-1}$ (Figure 6b,c). The thermal stability of the ${\mathrm{Zn}}^{2+}$‐linked gels was incrementally decreased by sequentially adding ${\mathrm{Br}}^{-}$ and ${\mathrm{Cl}}^{-}$ at a molar ratio of $n_{Br}/n_{Zn}$ = 5 and $n_{Cl}/n_{Zn}$ = 50, respectively. Zn(tpy)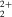 complexes, the dominant species before halide addition, gradually diminished in prevalence as externally added ${\mathrm{Br}}^{-}$ and ${\mathrm{Cl}}^{-}$ shifted the equilibrium toward non‐linking ${\mathrm{ZnBr}}_{4}^{2-}$ and ${\mathrm{ZnCl}}_{4}^{2-}$ complexes (Figure 6a, top). As a result, SAXS S(q) of the sample with ${\mathrm{Br}}^{-}$ showed reversible gelation with T_gel_ between 30 and 120 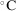, whereas the subsequent addition of a stronger coordinating halide, ${\mathrm{Cl}}^{-}$, restored a fully dispersed state even at 30 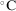 (Figure 6b). The colloidal phase behavior was further modulated in the reverse direction by introducing metal ions with stronger binding affinities to terpyridine. Although chloride remained in excess relative to the total metal ion concentration, the addition of ${\mathrm{Co}}^{2+}$ at lower temperature induced the formation of orange‐colored nanocrystal gels, characteristic of Co(tpy)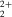 linkages, due to the its stronger coordination to terpyridine.^[^ ^9^ ^]^ At 120 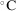, however, this ${\mathrm{Co}}^{2+}$‐linked gel transitioned into a free‐flowing dispersion without orange coloration, indicating a diminished prevalence of Co(tpy)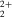 links at elevated temperature. Finally, addition of ${\mathrm{Ni}}^{2+}$, the strongest linker in the series, resulted in persistent aggregation across the thermal cycle, consistent with the formation of thermally stable Ni(tpy)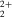 links. Notably, the gel lost its orange color as terpyridine preferentially formed more stable (colorless) coordination complexes with ${\mathrm{Ni}}^{2+}$ than with ${\mathrm{Co}}^{2+}$ (Figure 6a).

Modulating the nanocrystals' phase behavior enabled reversible switching between distinct infrared optical modes (Figures 6c and S14). Depending on whether the nanocrystals formed stable dispersions, thermoreversible gels, or thermally stable gels over the thermal cycle, their optical responses reversibly switched among three optical modes, exhibiting high–high, low–high, or low–low $\omega_{\text{LSPR}}$ at the two temperature setpoints, respectively. The high $\omega_{\text{LSPR}}$ values observed for free‐flowing dispersions were consistent at approximately 4800 ${\mathrm{cm}}^{-1}$ across all optical modes, as expected from identical plasmonic nanocrystals in a well‐dispersed state without interparticle LSPR coupling. In contrast, the low $\omega_{\text{LSPR}}$ values observed for gels were tunable through local gel coarsening. For example, compared to pure ${\mathrm{Zn}}^{2+}$‐linked gels at 30 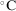, adding ${\mathrm{Br}}^{-}$ resulted in an increase in SAXS‐derived ΔS from 2.3 to 2.5 and a decrease in gel $\omega_{\text{LSPR}}$ from 4600 ${\mathrm{cm}}^{-1}$ to below 4400 ${\mathrm{cm}}^{-1}$. Altogether, these results demonstrate that sequential modulation of competitive metal–ligand equilibria provides a robust and reversible strategy to program nanocrystals' phase behavior and their structure‐dependent properties.

## Conclusion

Our findings establish competitive metal–ligand equilibria as a general and modular approach for directing nanocrystal gelation and tuning local gel structure, circumventing the need for intricate linker or ligand synthesis. The cumulative and hierarchical influence of multiple metal and halide ions enabled overwriting the effects of weaker coordination species, giving a rise to sequential modulation of ITO nanocrystals' phase behavior and their structure‐dependent infrared optical response. We envision that this strategy can extend to any coordination‐driven assembly, including quantum dot gels^[^ ^45^ ^]^ and metallopolymers,^[^ ^46^ ^]^ providing a versatile platform for designing responsive soft materials. Coupling this strategy with chemical fueling, where the concentrations of metal or competing ligands are dynamically varied, may further unlock pathways toward transient coordination assembly, allowing for temporal programming of material properties. Employing ligands that can form metal coordination links with greater geometric and stoichiometric diversity than terpyridine, such as (bi)pyridine and catechol, could also significantly enrich the structural and functional landscape of coordination‐driven assemblies. Lastly, direct quantification of the prevalence of different metal coordination species, similar to the spectroscopic analysis of linkages in ${\mathrm{Co}}^{2+}$‐linked gels,^[^ ^9^ ^]^ could provide generalized, quantitative designer rules for modulating local gel coarsening and tuning T_gel_.

## Author Contributions

J.K. and D.J.M. conceived the idea and designed the research. D.J.M., T.M.T., and E.V.A. guided the research. J.K., J.Lee and J.Liu performed nanocrystal synthesis, gelation and characterization. D.Q. performed kMC and MPM simulations. D.L.C. and J.D.O. synthesized and characterized ligands. M.W.B. conducted ICP‐OES analysis. J.K., D.J.M., D.Q., T.M.T., D.L.C., and J.Lee wrote the manuscript with input from all authors.

## Conflict of Interests

The authors declare no conflict of interest.

## Supporting information

Supporting Information

## Data Availability

The data supporting the findings of the manuscript is available upon a reasonable request from the corresponding authors.
