# Supplementary material for: Colloidal Phase Control in Plasmonic Metal Oxide Nanocrystals via Competitive Metal–Ligand Equilibria
Source: Angew Chem Int Ed Engl. 2025 Nov 4;64(52):e18965. doi: 10.1002/anie.202518965 (PMC12723473; doi:10.1002/anie.202518965)
Supplement: Supplementary file 1 — Supporting Information [file ANIE-64-e18965-s001.pdf]

Supporting Information

Colloidal Phase Control in Plasmonic Metal Oxide Nanocrystals via Competitive Metal–Ligand Equilibria

Jiho Kang,[†] Dingwen Qian,[†] Jayoon Lee,[‡] Diana L. Conrad,[‡] Jessica D. Oberlander,[‡] M. Wren Berry,[‡] Jeffrey Liu,[†] Eric V. Anslyn,[*,‡] Thomas M. Truskett,[*,¶,§,†] and Delia J. Milliron[*,¶,†,‡]

†*McKetta Department of Chemical Engineering, University of Texas at Austin, 200 E Dean Keeton St, Austin, Texas 78712, United States*

‡*Department of Chemistry, University of Texas at Austin, 2506 Speedway, Austin, Texas 78712, United States*

¶*Department of Chemical Engineering, University of Michigan, Ann Arbor, MI 48109, United States*

§*Biointerfaces Institute, University of Michigan, Ann Arbor, MI 48109, United States*

E-mail: anslyn@austin.utexas.edu; truskett@umich.edu; milliron@umich.edu

Contents

1. Synthetic Details

1.1 Tin-doped Indium Oxide ($Sn:In_2O_3$, ITO) Nanocrystals 3

1.2 Terpyridine-terminated Ligand (TL) 4

2. Instrumentation

2.1 UV-Vis-NIR Spectroscopy 6

2.2 Small-Angle X-ray Scattering (SAXS) 6

2.3 Scanning Transmission Electron Microscopy (STEM) 7

2.4 Fourier Transform Infrared (FTIR) Spectroscopy 7

2.5 Inductively Coupled Plasma-Optical Emission Spectroscopy (ICP-OES) 7

2.6 ^{1}H Nuclear Magnetic Resonance Spectroscopy (^{1}H NMR) 8

2.7 Thermogravimetric Analysis (TGA) 8

3. Experimental Methods

3.1 Surface Functionalization of Nanocrystals with TL 9

3.2 Ligand Quantification 9

3.3 Preparation of Nanocrystal Gels 11

4. Theoretical arguments and numerical methods

4.1 Kinetic Monte Carlo Simulation of the Gelation Process 12

4.2 Relationship between Bond Strength and Bond Liability 12

4.3 Renormalization of the Crawling Time in the Kinetic Monte Carlo Simulations .. 13

4.4 Electromagnetic simulations using the mutual polarization method 13

4. Supporting Figures and Tables 15

References 31

1. Synthetic Details

1.1 Tin-doped Indium Oxide ($Sn:In_2O_3$, ITO) Nanocrystals

Materials.

All chemicals were purchased commercially and used as received, without any additional purification. Indium(III) acetate (99.99%) was purchased from Strem. Tin(IV) acetate ($>$ 99.9%), oleic acid (90%, technical grade), and oleyl alcohol (98%) were purchased from Sigma-Aldrich. Hexane (99.8%) and ethanol (95.0%) were purchased from Fisher Chemical.

Synthesis.

ITO nanocrystals were synthesized on a Schlenk line with minor modifications to the synthetic protocols developed by the Hutchison group.[S1] Tin(IV) acetate (0.525 mmol, 0.186 g) and indium(III) acetate (9.975 mmol, 2.912 g) were added to a 50 mL round bottom flask and dissolved in 21 mL of oleic acid with stirring to prepare a solution of 0.5 M metal precursor. The precursor solution was degassed under vacuum, first at room temperature for 10 minutes and then at 90 °C for 1 hour. The temperature was subsequently raised to 150 °C and maintained with flowing N_2 for 3 hours. Meanwhile, 13 mL oleyl alcohol was added to a separate 100 mL round bottom flask and heated to 290 °C with stirring under N_2 flow. 6mL of the metal precursor solution was injected dropwise to the heated oleyl alcohol using a syringe pump at a rate of 0.2 mL/min. The reaction mixture was allowed to react for an additional 5 minutes after the injection and then cooled to room temperature by removing the flask from the heating mantle. Finally, the synthesized ITO nanocrystals were washed five times by flocculating with ethanol, centrifuging, and redispersing in neat hexane. Three batches of nanocrystals were synthesized from the metal precursor solution and subsequently combined for further characterization.

1.2 Terpyridine-terminated ligand (TL)

Materials.

Unless otherwise specified, all reagents were used as received without additional purification.

(1) Solid-Phase Peptide Synthesis (SPPS)

Fmoc-Asp(OtBu)- Wang Resin (0.36 mmol/g, mesh 100-200), Fmoc-Asp(OtBu)-OH, and *N,N'*-diisopropylcarbodiimide (99.5 %) were purchased from Sigma Aldrich. Fmoc-NH-PEG_2-CH_2CH_2COOH (99.9 %) was purchased from PurePEG, LLC. AstaTech, Inc. supplied the Fmoc-Azido-L-Lysine (99.9 %). Fmoc-L-Phe(2-trifluoromethyl)-OH (≥99 %) and ethyl(hydroxyamino) cyanoacetate were purchased from Chem-Impex Int'l Inc. ACS grade acetic anhydride, and sequencing grade *N,N*-dimethylformamide (DMF) were purchased from Fisher Scientific. Piperidine (99 %) was purchased from Alfa Aesar.

(2) Solid-Phase Copper-Catalyzed Azide-Alkyne Cycloaddition (SP-CuAAC)

Copper(I) iodide (99.99 %) and (+)-sodium L-ascorbate (98 %) were obtained from Sigma-Aldrich. Tris(benzyltriazolylmethyl)amine (TBTA) and the terpyridine-based alkyne were synthesized using previously published procedures.[S2,S3]

(3) Resin Cleavage

Trifluoroacetic acid was purchased from Chem-Impex Int'l Inc. Nanopure water was provided by the University of Texas at Austin. Triisopropylsilane (98 %) was purchased from Aldrich Chemistry. Nanopure water used in all procedures was provided by the University of Texas at Austin.

Synthesis.

A full description of the synthetic procedure and product characterization can be found

in our previous works.[S3–S5]

Resin bound peptides were synthesized on a 0.25 mmol scale utilizing standard Fmoc and tBu protection strategies via microwave assisted SPPS using a CEM Liberty Blue Automated Peptide Synthesizer. Couplings to the Wang resin were carried out using DIC and Oxyma at 90 °C for 110 s, adding monomers of Fmoc-Asp(OtBu)-OH, Fmoc-NH-PEG$_2$-CH$_2$CH$_2$COOH, and Fmoc-Azido-L-Lysine. Fmoc deprotection was achieved with 20 % piperidine in DMF in two steps: (1) 75 °C, 155 W, 15 s and (2) 90 °C, 155 W, 50 s. The resin was washed sequentially with DMF, DCM, MeOH then dried.

Terpyridine functionalization was achieved via SP-CuAAC under inert atmosphere. The resin was reacted with the terpyridine alkyne (1.3 eq), TBTA (0.4 eq), sodium ascorbate (0.4 eq), and CuI (0.01 eq) in 10 mL of 4:1 (vol/vol) DMF/H_2O and agitated for 72 hours.

Post-reaction, the peptide was again washed with DMF/DCM/MeOH and dried. The ligand was cleaved from the resin using 95:2.5:2.5 (vol/vol) TFA/H_2O/TIPS for 4 hours. The crude product was precipitated with diethyl ether, dried, then purified using high performance liquid chromatography (HPLC) (10-95 % MeOH in H_2O, 0.1 % volume formic acid, 50 min binary gradient). Product peaks were identified using liquid-chromatography mass spectrometry (LCMS). MeOH was removed via rotary evaporation and the remaining aqueous solution was lyophilized, yielding a pale yellow powder.

TL: Yield 24%; HRMS-ESI (m/z): $[M]^+$ calc. for $C_{53}H_{65}N_{11}O_{15}$ $[M+H]^+_{Theoretical}$ = 1096.4734, $[M+H]^+_{Observed}$ = 1096.4691, Target Mass Error = -0.0043 ppm.

2. Instrumentation

2.1 UV-Vis-NIR Spectroscopy

UV-Vis-NIR spectra were collected using an Agilent-Cary 5000 spectrophotometer. The LSPR absorption spectra of diluted dispersions (0.2 mg/mL) of oleate-ITO in tetrachloroethylene and TL-ITO in DMF were obtained using a near infrared quartz cuvette with a 2 mm pathlength (Spectrocell). The LSPR response of nanocrystal gel assemblies at varying temperatures was monitored using a temperature-controlled liquid cell (Harrick Scientific). The gels were initially converted to free-flowing dispersions by heating them on a hotplate at 120 °C and then transferred into the cell consisting of two 2-mm-thick CaF_2 windows separated by a 56-μm-thick spacer. The sample was maintained at each target temperature for 5 minutes before collecting the spectra. The LSPR response of nanocrystal gel assemblies at extended aging period (up to 1 week) was measured using home-made glass cells. Two glass slides (25 mm $\times$ 25 mm), one with two pre-drilled holes, were assembled into a sandwich cell using a 60-μm-thick thermoplastic sealant (Surlyn, Solaronix SA). Samples were injected into the cell through the holes, which were then sealed with Kapton tape. The homemade cells were stored in a nitrogen box unless their absorption spectra were being collected.

2.2 Small-Angle X-ray Scattering (SAXS)

SAXS patterns were collected in transmission configuration using the SAXSLAB Ganesha instrument at the Texas Materials Institute, University of Texas at Austin. All samples were contained in flame-sealed glass capillaries (Charles Supper Company, Inc.). Silver behenate standard was employed to calibrate sample-to-detector distance (ca. 1 m). The obtained SAXS patterns were processed using Igor Pro 8-based Irena and Nika software package. The diameter of the inorganic nanocrystal core was determined by fitting the scattering pattern of oleate-ITO dispersed in hexane using a spheroid form factor. The SAXS structure factors $S(q)$ were calculated by dividing the measured intensity profile by the scattering pattern of a dilute dispersion of oleate-ITO in hexane.[S2] A temperature-controlled stage (Linkam

Scientific) was used for SAXS measurements at varying temperatures, with a homemade gold cover film to improve thermal contact between the cell and capillary. The capillary was left at each target temperature for 3 minutes before collecting their scattering patterns.

2.3 Fourier Transform Infrared (FTIR) Spectroscopy

Transmission FTIR spectra of oleate-ITO, TL, and TL-ITO were acquired using a Bruker Vertex 70 spectrometer with a resolution of 4 cm^{-1}. Samples were prepared by drop-casting dilute dispersions of oleate-ITO in hexane, TL in DMF, or TL-ITO in DMF onto double-side polished silicon wafers, followed by drying on a hotplate at 60 °C.

2.4 Scanning Transmission Electron Microscopy (STEM)

Electron microscopy images of as-synthesized ITO nanocrystals were obtained using a Hitachi S5500 scanning transmission electron microscope (STEM), with an accelerating voltage of 30 kV. The sample was prepared by dropcasting a dilute dispersion (0.2 mg/mL) of oleate-ITO in hexane onto Cu 400-mesh TEM grids with carbon support films, followed by drying under vacuum overnight.

2.5 Inductively Coupled Plasma-Optical Emission Spectroscopy (ICP-OES)

Sn doping concentration and volume fraction of ITO nanocrystal cores in the stock solution was determined by measuring Sn and In concentrations of the digested solution using an Agilent 5800 ICP-OES. First, a known volume of oleate-ITO in hexane was added to a 15 mL falcon tube and dried into a pellet in ambient condition. The pellet was then digested in aqua regia for 3 days and subsequently diluted with Milli-Q water to a final acid concentration of 2 vol.%. Standard solutions with varying Sn and In content were prepared by diluting the standard solutions with 2 vol.% nitric acid. Sn at.% was calculated by dividing the measured Sn concentration by the combined metal concentration (Sn + In). The ITO volume fraction in the stock solution was calculated by first converting the measured metal

content to volume using the density of ITO (7.14 g/cm^3), and then dividing it by the volume of stock used for ICP-OES measurement.

2.6 ^{1}H Nuclear Magnetic Resonance Spectroscopy (^{1}H NMR)

^{1}H NMR spectra of TL–ITO and TL were recorded on a Bruker 3M 500 NMR spectrometer at 500 MHz using DMSO–d_6 as the solvent. NMR spectra were acquired with a relaxation delay of 10 s, an acquisition time of 3.276 s, a total of 128 scans, a pulse angle of 90 °, and their chemical shifts were referenced to DMSO-d_6 peaks. All NMR data were processed using MNova software.

2.7 Thermogravimetric Analysis (TGA)

TGA of TL-ITO was performed using a Mettler Toledo TGA 2 instrument under an air atmosphere. The TGA sample was prepared by placing a stock solution of TL-ITO into an alumina crucible and drying it on a hotplate at 80 °C prior to measurement. The heating protocol consisted of two stages. First, the temperature was ramped under air from 25 °C to 150 °C at a rate of 10 °C min^{-1} and and held isothermally at 150 °C for 60 min to ensure complete removal of residual solvent. The sample was then heated further under air to 600 °C at a rate of 2 °C min^{-1} to analyze the mass loss associated with the organic ligand shell.

3. Experimental Methods

3.1 Surface Functionalization of Nanocrystals with TL

Native oleate ligands on nanocrystal surface were partially replaced with TL via a direct ligand exchange. Oleate-ITO dispersed in hexane were flocculated using ethanol and centrifuged. After discarding the supernatant, the flocculated nanocrystals were dried in a N_2 flow to form a nanocrystal pellet. The pellet was then sonicated for 3 hours in a solution of 0.01 M TL in DMF. The initially cloudy solution became transparent within one hour of sonication. The TL-functionalized nanocrystals (TL-ITO) were left overnight to allow further ligand exchange. Finally, TL-ITO were flocculated by adding a 4:6 (v:v) mixture of ethanol and hexane and re-dispersed in neat DMF. This washing process was repeated once more before preparing the final stock solution of TL-ITO in DMF.

3.2 Ligand Quantification

Native oleate ligands on the surface of OA-ITO are partially replaced with TL through direct ligand exchange driven by mass action. The loading of remaining oleate ligands and bound TL on TL-ITO was quantified using Thermogravimetric analysis (TGA) and ^{1}H NMR. TGA was first performed on TL-ITO to estimate the total loading of surface ligands (i.e., oleate and TL combined) (Fig. S6). The ligand shell and inorganic nanocrystal core accounted for 13.6 wt.% and 86.4 wt.%, respectively. The molar concentration of nanocrystals was estimated from the SAXS-derived core diameter (12.1 nm) and the mass of the nanocrystal inorganic core, obtained by correcting the mass of dried OA-ITO pellets with the core mass fraction determined from TGA.

To quantify TL tethered to the nanocrystal surface, ^{1}H NMR spectra were collected for TL–ITO using an internal reference of known concentration. A stock solution of 1,3,5-trimethoxybenzene (0.1 M, in deuterated dimethyl sulfoxide (DMSO-d_6)) was prepared as an internal standard. TL–ITO (11.3 mg, corresponding to 2.4 nmol of particles) was precipitated using a mixture of 4:6 (v/v) ethanol:hexane mixture and collected by centrifugation (10800

rpm, 5 min). The supernatant was discarded, and the resulting pellet was gently dried under a nitrogen flow. To remove residual non-deuterated solvents, deuterated ethanol was added to the pellet, followed by sonication for 5 minutes and brief centrifugation (10800 rpm, 1 min). The supernatant was carefully removed, and the pellet was dried under a nitrogen flow. This washing process was repeated two additional times. After the final wash, the pellet was dried under nitrogen and redispersed in 693 μL DMSO–d_6 along with 7 μL of internal standard stock solution, yielding final concentration of 1 mM for the internal standard and 3.5 μM for TL-ITO. The loading of TL per nanocrystal (N_{TL}) was quantified by comparing the integrated intensity of the singlet at 6.09 ppm (3 H), corresponding to the aromatic protons of 1,3,5-trimethoxybenzene, with that of the broad resonances of TL between 7 and 9 ppm (20 H), yielding $N_{TL} = 190$.

The loading of oleate ligands on TL-ITO was estimated as follows. First, the mass of a single ITO nanocrystal inorganic core (m_{NC}) with 12.1 nm diameter is:

$$m_{NC} = V_{NC} \times \rho_{NC} = 9.338 \times 10^{-19}\, cm^3 \times 7.16\, g\, cm^{-3} = 6.69 \times 10^{-18}\, g$$

where V_{NC} and ρ_{NC} are the nanocrystal's volume and density, respectively. The mass of ligand shell per nanocrystal (m_{Ligand}) is:

$$m_{Ligand} = m_{NC} \times \frac{\text{wt.\% ligand}}{\text{wt.\% nanocrystal core}} = 6.69 \times 10^{-18}\, g \times \frac{13.6\,\text{wt.\%}}{86.4\,\text{wt.\%}} = 1.05 \times 10^{-18}\, g$$

Mass of TL on a single ITO nanocrystal (m_{TL}) is:

$$m_{TL} = N_{TL} \times \frac{MW_{TL}}{n_A} = 190 \times \frac{1096.47\, g\, mol^{-1}}{6.022 \times 10^{23}\, mol^{-1}} = 3.46 \times 10^{-19}\, g$$

where MW_{TL} and n_A are the molecular weight of TL and Avogadro's number, respectively. The mass of bound oleate ligands per nanocrystal (m_{OA}) is obtained by subtracting TL's

contribution from m_{Ligand}:

$$m_{OA} = m_{Ligand} - m_{TL} = 1.05 \times 10^{-18}\, g - 3.46 \times 10^{-19}\, g = 7.1 \times 10^{-19}\, g$$

Finally, the number of oleate ligands per nanocrystal (N_{OA}) is:

$$N_{OA} = m_{OA} \times \frac{n_A}{MW_{OA}} = 7.1 \times 10^{-19}\, g \times \frac{6.022 \times 10^{23}\, mol^{-1}}{282.46\, g\, mol^{-1}} = 1510$$

Therefore, the number of oleate and TL ligands per nanocrystal is estimated to be 1510 and 190, respectively, giving a ratio of 8:1 (oleate:TL).

3.3 Preparation of Nanocrystal Gels

Nanocrystal gel assemblies were prepared by mixing stock solutions, all of which were prepared in anhydrous DMF within a nitrogen glove box. Specifically, stocks of 50.0 mg/mL (0.7 vol.%) TL-ITO, 0.5 M tetrabutylammonium halide (TBAX, X = Cl, Br, or I), 0.02 M metal dichloride (MCl_2, M = Mn, Fe, Co, Ni, Cu, or Zn), and neat DMF were mixed to prepare gels. The final concentrations of ITO nanocrystals and MCl_2 were fixed at 1.7 μM (0.1 vol.%) and 2 mM, respectively. The final concentration of TBAX was varied between 0 and 496 mM depending on samples.

4. Theoretical Arguments and Numerical Methods

4.1 Kinetic Monte Carlo Simulation of the Gelation Process

Initial configurations of nanocrystals are prepared by randomly assigning positions to 10000 spherical particles in a cubic simulation box with periodic boundary conditions. The characteristic collision time of single particles through the diffusion process is $\tau_{\text{collision}} = 1/(8\pi Ddn)$, where D is the diffusion coefficient of the single particles, d is the distance at which particles begin to form bonds, and n is the number density of particles.[S6] Since the unit time of the simulation is $\tau_0 = \Delta x^2/(12D)$,[S7] the characteristic collision time is related to the unit time by $\tau_{\text{collison}} = 3\tau_0/(2\pi dn\Delta x^2)$, where Δx is the step size of the Monte Carlo movement. In the simulations, particles form bonds when they collide, which renormalizes the time scale by setting $\tau_{\text{assoc}} = \tau_{\text{collision}}$. The crawling time is then related to the diffusion time scale by $\tau_{\text{crawl}} = \tau_{\text{collision}} A \exp[-\Delta G_{\text{bond}}/(k_{\text{B}}T)] = [3\tau_0/(2\pi dn\Delta x^2)]A\exp[-\Delta G_{\text{bond}}/(k_{\text{B}}T)]$, where A is a constant. The crawling time increases as the bond strength increases. In Fig. S17, we show the reason why increasing the crawling time leads to a more fractal structure.

4.2 Relationship between Bond Strength and Bond Lability.

When ligands can form strong metal coordination linkages with other ligands on the same nanoparticle (i.e., self-loops) or linker-mediated interparticle bridges, the driving force for nanoparticle association is entropic. Association of nanoparticles grafted with palindromic (self-complementary) DNA sticky sequences is an example of a similar system with interparticle bridges forming at the expense of self loops. Experiments and simulations of the latter system have established that association processes are favored by an increase in combinatorial entropy.[S8] However, it is not well understood what controls the kinetics of structural evolution of such associated nanoparticles, which is crucial for gel formation.

As a simplification, we envision the assembly process as involving two primary events in the strong association limit: (1) interparticle association and (2) crawling of nanoparticles around each other while they remain associated. Here, we consider the minimal association

event as limited by the rate of breaking two proximal self loops (one on each particle) to form two bridges. Crawling of an associated pair would require breaking one of the interparticle bridges and two more proximal self loops (one on each particle) so that a new bridge can be formed. Since crawling requires breaking one more bond than association, we expect the characteristic crawling time to be longer than the characteristic association time by a factor proportional to $\exp[-\Delta G_{\mathrm{bond}}/(k_{\mathrm{B}}T)]$, where ΔG_{bond} is the free energy change of bond formation, and k_{B} is Boltzmann's constant.

4.3 Renormalization of the Crawling Time in the Kinetic Monte Carlo Simulations

The characteristic collision time of single particles through the diffusion process is $\tau_{\mathrm{collision}} = 1/(8\pi Ddn)$, where D is the diffusion coefficient of the single particles, d is the distance at which particles begin to form bonds, and n is the number density of particles.[S6] Since the unit time of the simulation is $\tau_0 = \Delta x^2/(12D)$,[S7] the characteristic collision time is related to the unit time by $\tau_{\mathrm{collison}} = 3\tau_0/(2\pi dn\Delta x^2)$, where Δx is the step size of the random movement of the single particles. In the simulations, particles form bonds when they collide, which renormalizes the time scale by setting $\tau_{\mathrm{assoc}} = \tau_{\mathrm{collision}}$. The crawling time is then related to the diffusion time scale by $\tau_{\mathrm{crawl}} = \tau_{\mathrm{collision}} A \exp[-\Delta G_{\mathrm{bond}}/(k_{\mathrm{B}}T)] = [3/(2\pi dn\Delta x^2)] A \exp[-\Delta G_{\mathrm{bond}}/(k_{\mathrm{B}}T)]\tau_0$, where A is a constant. The crawling time increases as the bond strength increases. In Fig. S17, we show the reason why increasing the crawling time leads to a more fractal structure.

4.4 Electromagnetic simulations using the mutual polarization method. Dispersion and gel optical properties were simulated using the mutual polarization method (MPM), which is a coupled dipole method with high computational efficiency.[S9,S10] MPM leverages a matrix-free, spectrally accurate Ewald summation method to solve a set of linear

equations for coupled dipoles of the nanocrytals:

$$\mathbf{E}_0 = \Sigma_j \mathbf{M}_{ij}\mathbf{p}_j,\ \mathbf{M}_{ij} = \begin{cases} \dfrac{\mathbf{I}}{4\pi a^3 \varepsilon_m \alpha}, & i = j \\ \dfrac{\mathbf{I} - 3\hat{\mathbf{r}}\hat{\mathbf{r}}}{4\pi r^3 \varepsilon_m}, & i \neq j \end{cases}$$

where a is the radius of the optical cores, $\mathbf{E}_0$ is the external optical field, $\alpha = (\varepsilon_p - \varepsilon_m)/(\varepsilon_p + 2\varepsilon_m)$ is the dipole polarizability of the nanocrystals, ε_p is the particle permittivity and $\varepsilon_m = 2.04$ is the medium permittivity (DMF), $\mathbf{r}$ is the displacement vector between particle i and particle j, $r = |\mathbf{r}|$ and $\hat{\mathbf{r}} = \mathbf{r}/r$. After solving for the nanocrystal dipole moments in simulated configurations, the extinction spectra can be directly computed.[S9]

The ITO nanocrystals permittivity was modeled with a core-shell dielectric function[S11]

$$\varepsilon_p(\omega) = \varepsilon_s \frac{\varepsilon_c + 2\varepsilon_s + 2\eta_c(\varepsilon_c - \varepsilon_s)}{\varepsilon_c + 2\varepsilon_s - \eta_c(\varepsilon_c - \varepsilon_s)}, \varepsilon_s = \varepsilon_\infty, \varepsilon_c(\omega) = \varepsilon_\infty - \frac{\varepsilon_0 \omega_p}{\omega^2 + i\gamma\omega},$$

where ε_s is the permittivity of electron-deficient shell, ε_c is the permittivity of electron-rich core, η_c is the core volume fraction, ω is the light frequency, ε_0 is the vacuum permittivity, ω_p is the plasma frequency, γ is the damping coefficient, and $\varepsilon_\infty = 4\varepsilon_0$ is the high-frequency permittivity of ITO. The values of $\omega_p = 14181$ cm^{-1}, $\gamma = 1066$ cm^{-1} and $\eta_c = 0.912$ were derived from the experimental extinction spectrum of a nanocrystal dispersion, fitted using the heterogeneous ensemble Drude approximation (HEDA) model (Fig. S15 and Table S1).[S11]

4. Supporting Figures and Tables

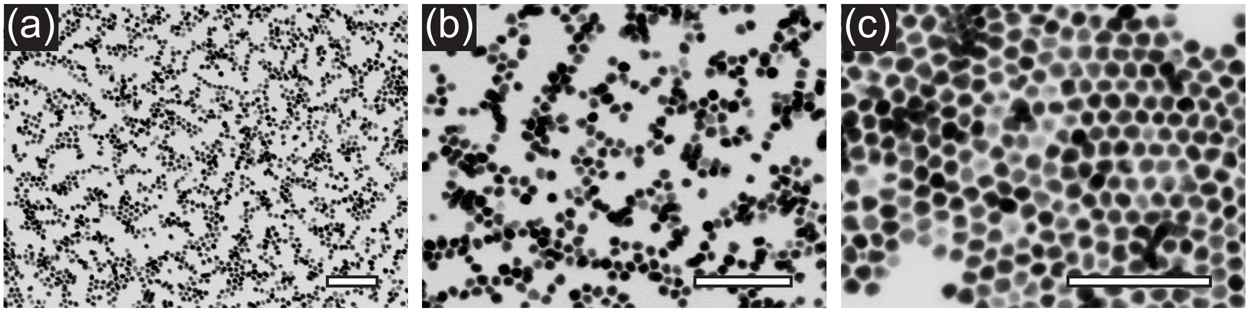

Figure S1: **STEM images of as-synthesized nanocrystals.** (a-c) Monodisperse, quasi-spherical ITO nanocrystals capped by native oleate ligands. Scale bars, 100 nm.

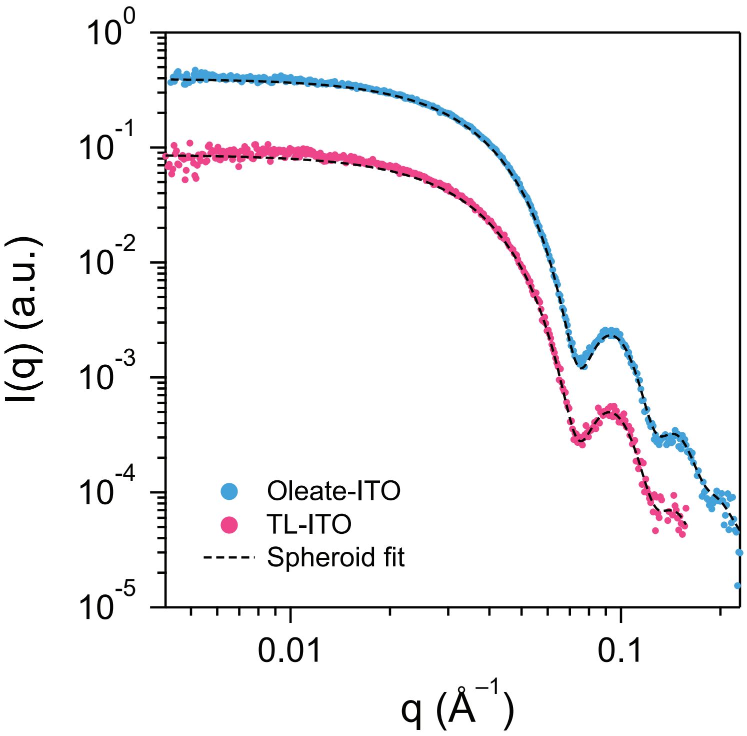

Figure S2: **SAXS patterns of ITO nanocrystals before and after TL functionalization.** SAXS patterns for dilute dispersions of oleate-ITO in hexane (blue solid dots) and TL-ITO in DMF (red solid dots). The nanocrystal diameter was determined to be 12.1 $\pm$ 1.2 nm from spheroidal form factor fits (dashed curves) and remained unchanged after ligand exchange.

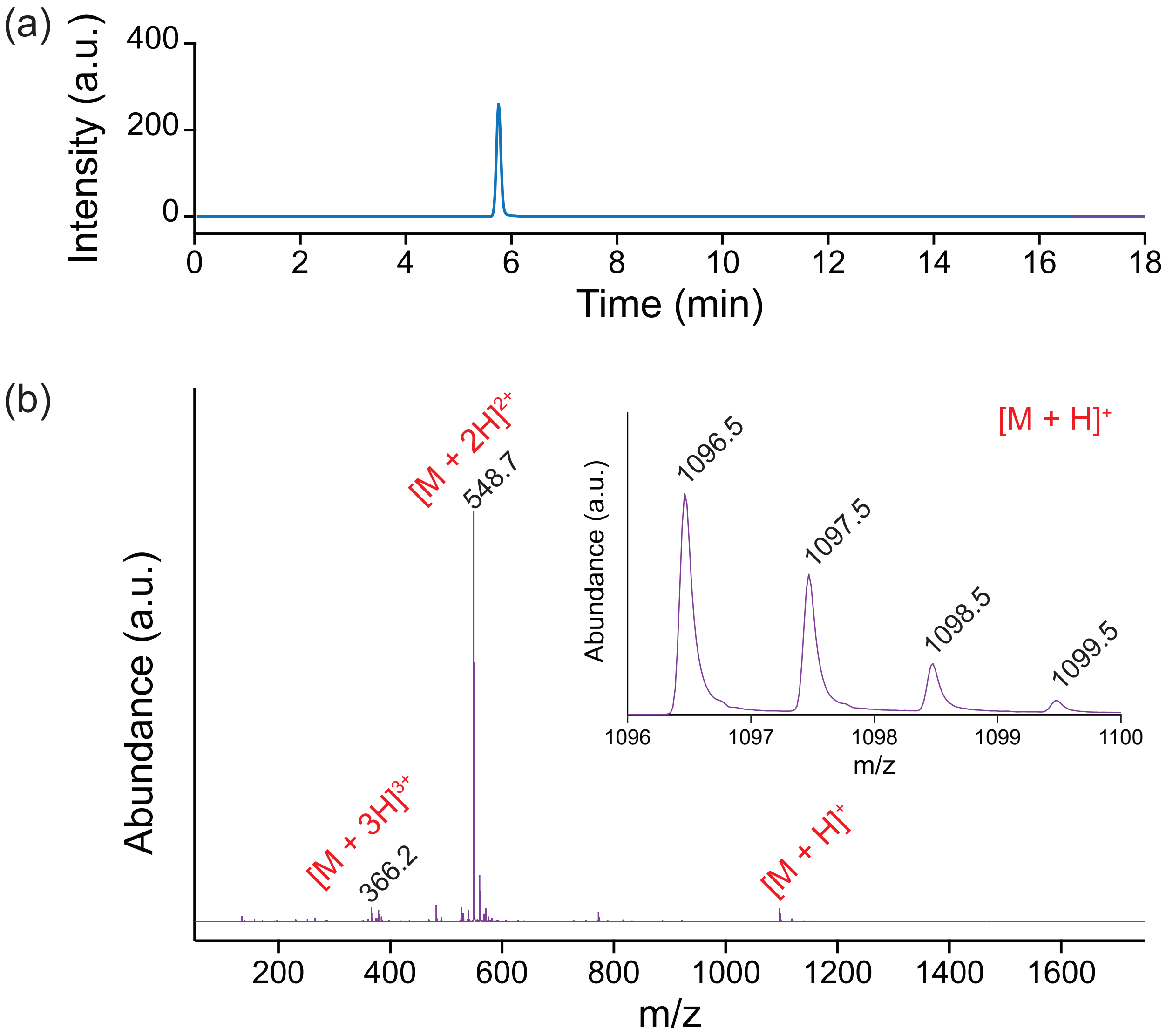

Figure S3: **Characterization of purified TL.** (a) LCMS trace and (b) corresponding mass spectrum of TL. The inset in (b) shows a magnified view.

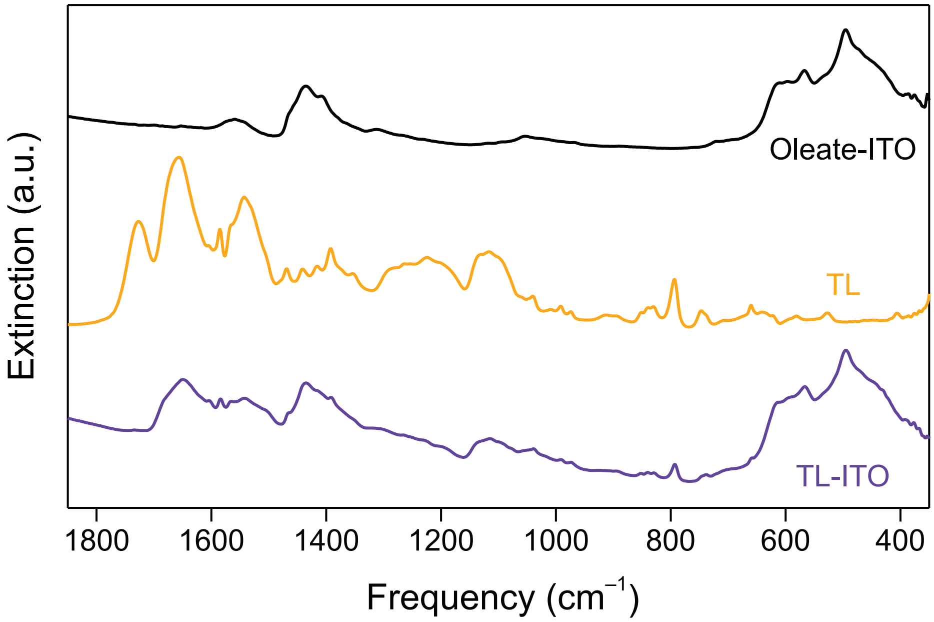

Figure S4: **FTIR spectra of TL, TL-ITO, and oleate-ITO.** Unlike oleate-ITO, TL-ITO presents a unique spectral fingerprint that reflects the presence of TL tethered to nanocrystal surface: 1728 cm^{-1} v(C=O) of carboxylic acid, 1650∼1660 cm^{-1} carbonyl and amide group, 1603 cm^{-1} v(C=C) of aromatic ring, 1585 cm^{-1} δ(N-H) of amide(II), 1566 cm^{-1} v(C=N) of pyridine, 1416 cm^{-1} v_s(C-O) of PEG, 1040 cm^{-1} v_{assoc}(C-O) of PEG, 794 cm^{-1} v(C-C) between pyridine rings. Note that the absence of the C=O stretching band at 1726 cm^{-1}, characteristic of carboxylic acid groups, in TL-ITO compared to TL indicates that the ligands are coordinated to the nanocrystal surface as carboxylates.

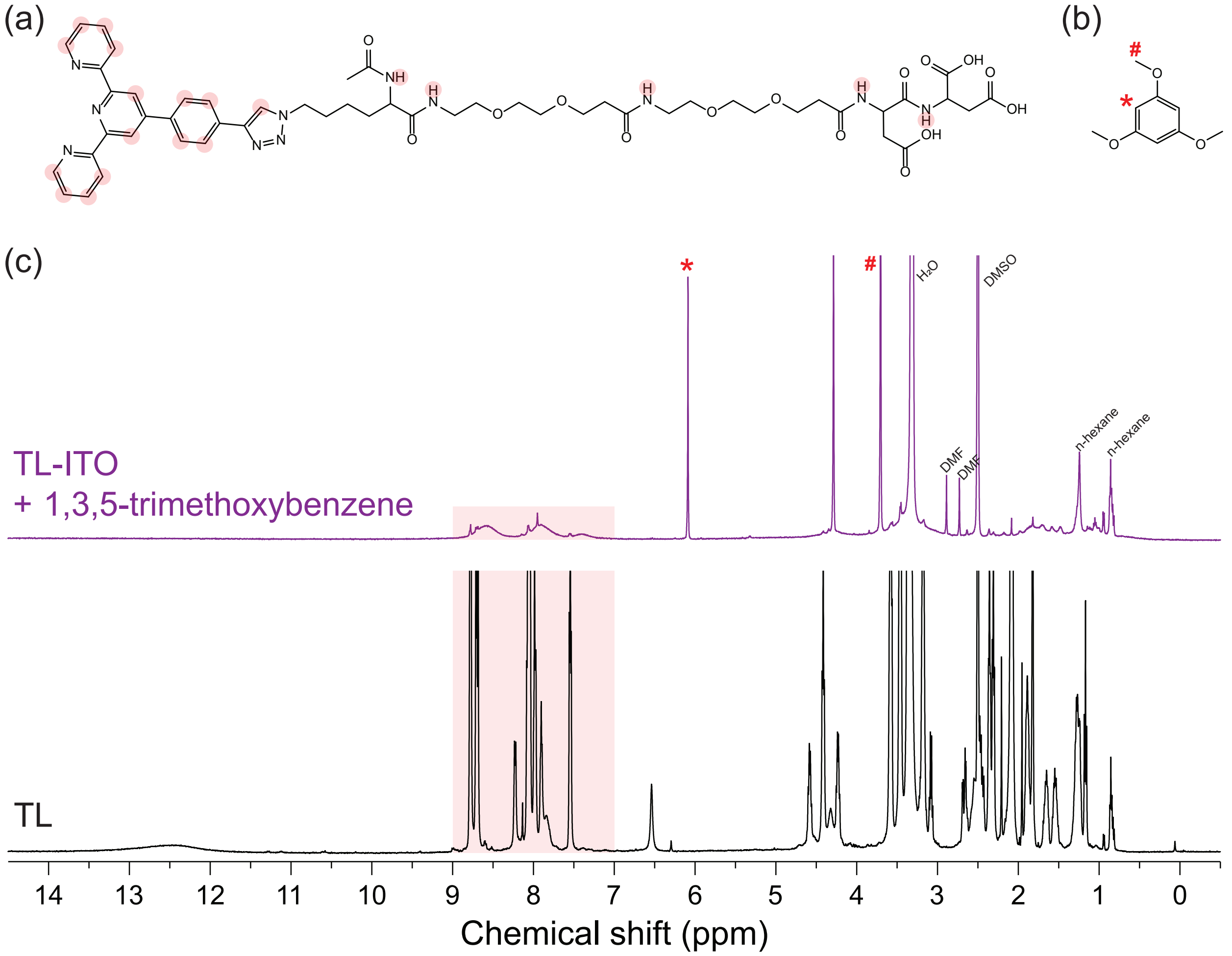

Figure S5: **Quantification of TL tethered to nanocrystals using ^{1}H NMR.** (a) Chemical structure of TL, with conjugated protons and amide protons indicated by red circles. (b) Chemical structure of 1,3,5-trimethoxybenzene, an internal reference. (c) ^{1}H NMR spectra of free TL (bottom) and a mixture of TL-ITO (4 µM) with 1,3,5-trimethoxybenzene (1 mM) in DMSO-d6. The terpyridine and amide peaks (7–9 ppm) are substantially broadened in TL-ITO, indicating reduced molecular mobility and heterogeneous chemical environments near the nanocrystal surface.[S12] The broad peak at 12.5 ppm, corresponding to the carboxylic acid of unbound TL, is absent in TL-ITO, consistent with ligand attachment to nanocrystals through carboxylate group. The concentration of bound TL was determined by comparing the integral intensity of the sharp singlet from the internal reference at 6.09 ppm (3H, indicated by *) to that of the broadened bands of TL (7–9 ppm, 20H, red shaded area). The average TL per nanocrystal was calculated by dividing the concentration of bound TL by that of nanocrystals. The molar concentration of ITO nanocrystals was determined by weight.

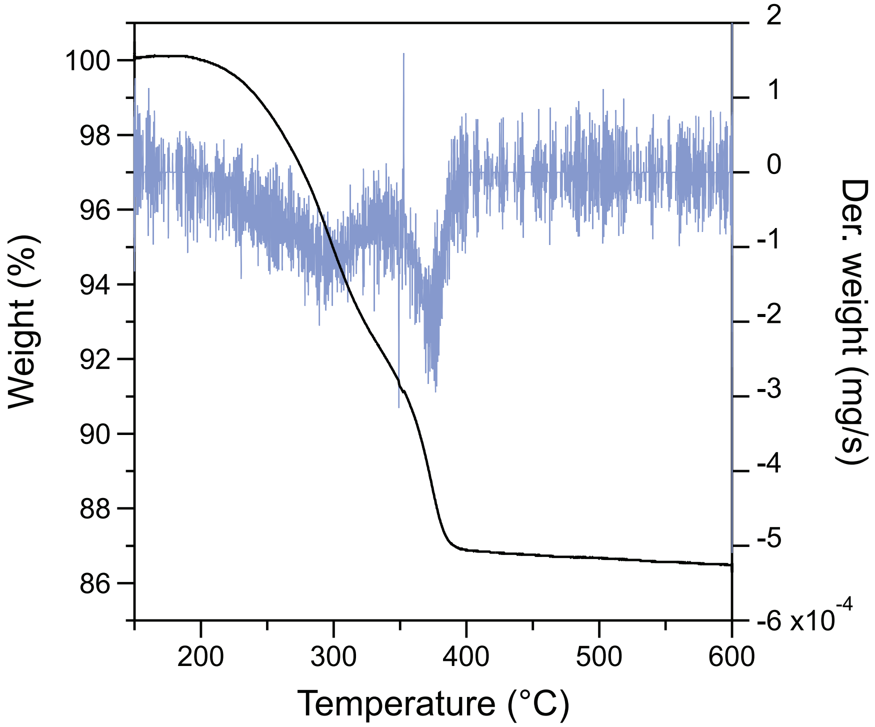

Figure S6: **Thermogravimetric analysis (TGA) curve of TL-ITO.** Shown are the residual weight (black) and derivative weight (blue) as functions of temperature from 150 to 600 °C, measured at a heating rate of 2 °C min^{-1} under air flow. Major thermal decomposition occurs between 200 °C and 400 °C, leaving a final weight residue of 86.4 % at 600 °C.

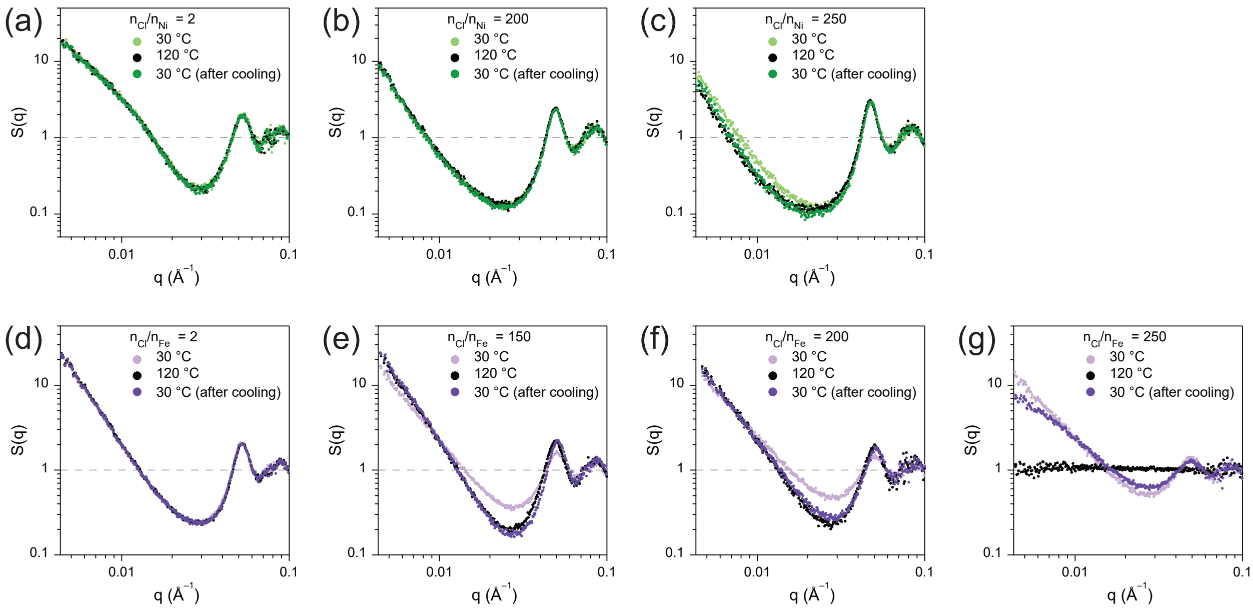

Figure S7: **Temperature-dependent phase behavior of TL-ITO solutions with Ni^{2+} or Fe^{2+} at various chloride ion concentrations.** (a-c) SAXS structure factor $S(q)$ of samples containing TL-ITO, $NiCl_2$, and TBACl with chloride-to-nickel(II) molar ratios n_{Cl}/n_{Ni} of (a) 2, (b) 200, and (c) 250. (d-g) SAXS $S(q)$ of samples containing TL-ITO, $FeCl_2$, and TBACl with chloride-to-iron(II) molar ratios n_{Cl}/n_{Fe} of (d) 2, (e) 150, (f) 200, and (g) 250. SAXS patterns were collected sequentially at 30 °C, 120 °C, and again at 30 °C, with each measurement performed after 5 min of thermal equilibration at the target temperature.

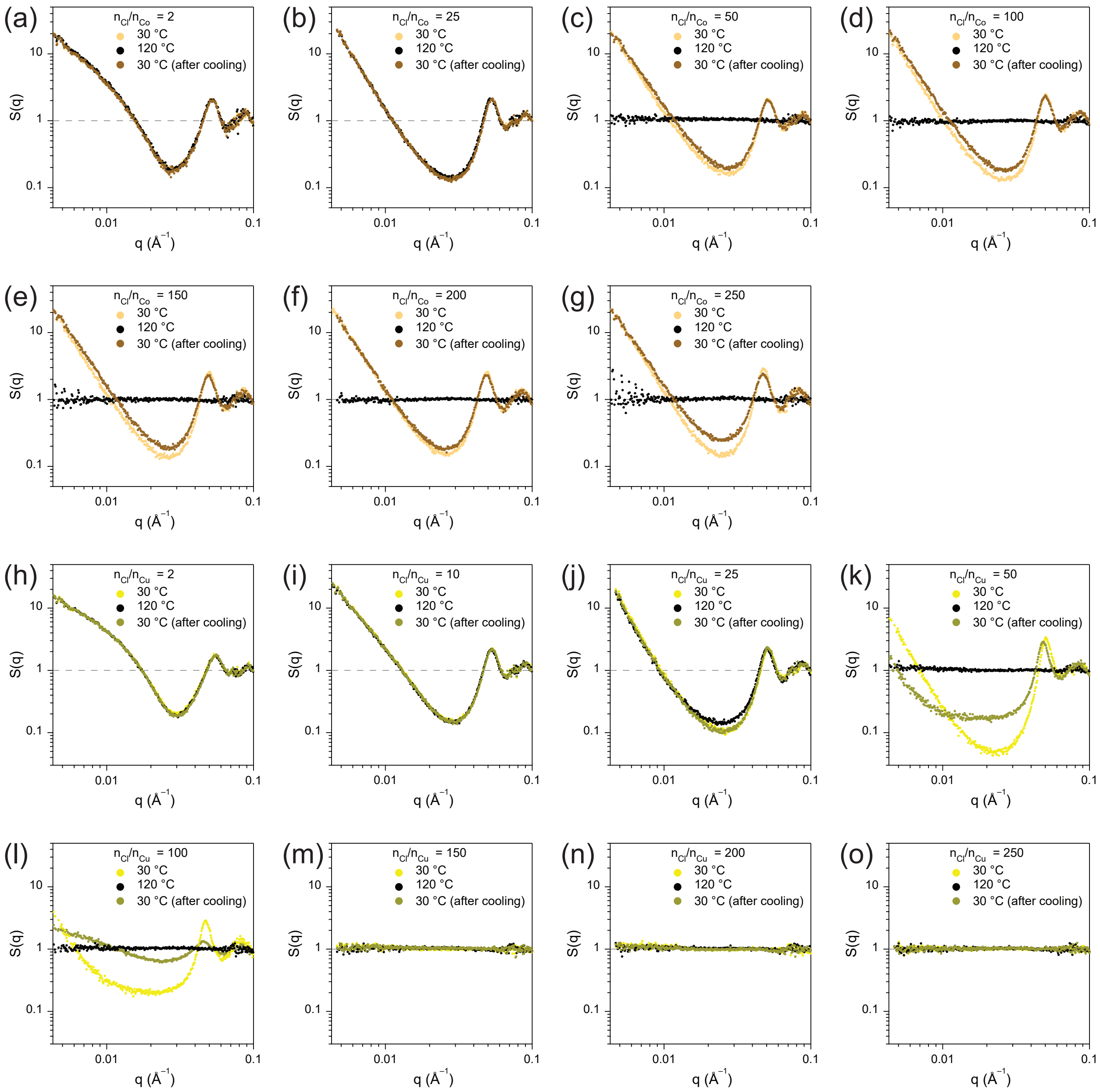

Figure S8: **Temperature-dependent phase behavior of TL-ITO solutions with Co^{2+} or Cu^{2+} at various chloride ion concentrations.** (a-g) SAXS structure factor $S(q)$ of samples containing TL-ITO, $CoCl_2$, and TBACl with chloride-to-cobalt(II) molar ratios n_{Cl}/n_{Co} of (a) 2, (b) 25, and (c) 50, (d) 100, (e) 150, (f) 200, and (g) 250. (h-o) SAXS $S(q)$ of samples containing TL-ITO, $CuCl_2$, and TBACl with chloride-to-iron(II) molar ratios n_{Cl}/n_{Cu} of (h) 2, (i) 10, (j) 25, (k) 50, (l) 100, (m) 150, (n) 200, and (o) 250. SAXS patterns were collected sequentially at 30 °C, 120 °C, and again at 30 °C, with each measurement performed after 5 min of thermal equilibration at the target temperature.

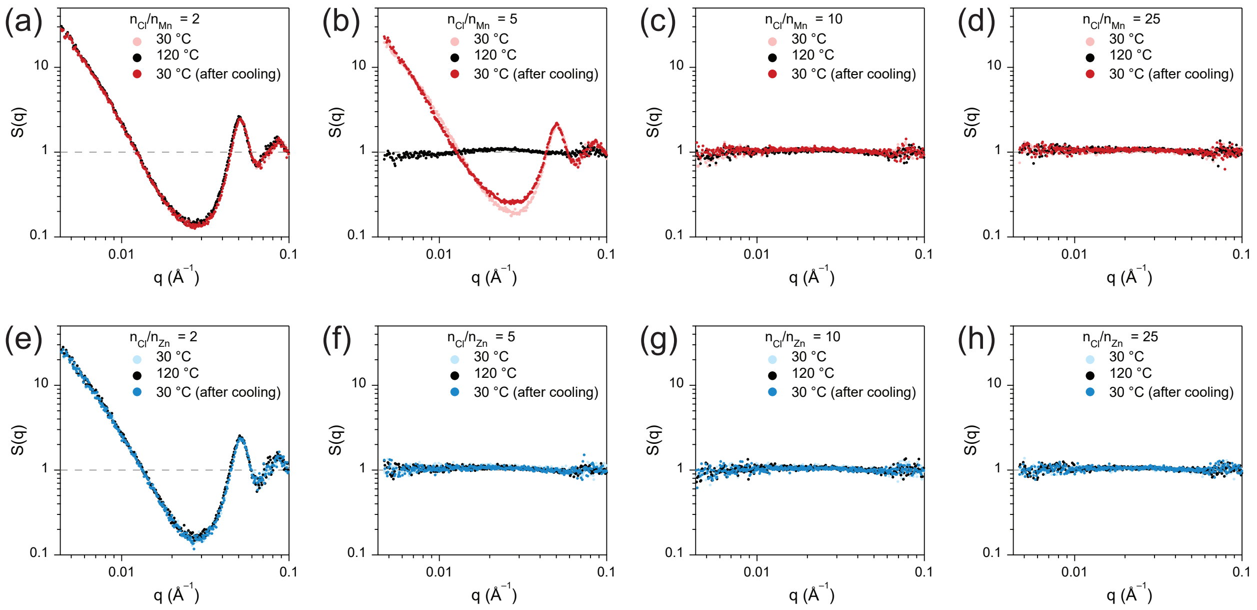

Figure S9: **Temperature-dependent phase behavior of TL-ITO solutions with Mn^{2+} or Zn^{2+} at various chloride ion concentrations.** (a-d) SAXS structure factor $S(q)$ of samples containing TL-ITO, $MnCl_2$, and TBACl with chloride-to-manganese(II) molar ratios n_{Cl}/n_{Mn} of (a) 2, (b) 5, (c) 10, and (d) 25. (e-h) SAXS $S(q)$ of samples containing TL-ITO, $ZnCl_2$, and TBACl with chloride-to-zinc(II) molar ratios n_{Cl}/n_{Zn} of (e) 2, (f) 5, (g) 10, and (h) 25. SAXS patterns were collected sequentially at 30 °C, 120 °C, and again at 30 °C, with each measurement performed after 5 min of thermal equilibration at the target temperature.

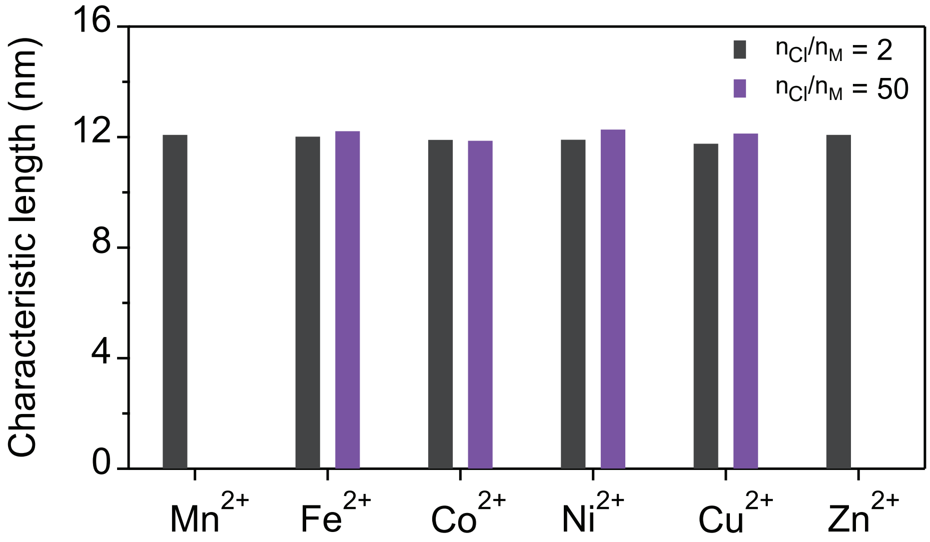

Figure S10: **Interparticle spacing of nanocrystal assemblies.** The characteristic length ($2\pi/q_{peak}$) calculated from the primary peak position q_{peak} of SAXS structure factor $S(q)$ for TL-ITO gels assembled with various divalent metal ions (M = Mn, Fe, Co, Ni, Cu, or Zn) at chloride-to-metal molar ratios n_{Cl}/n_M of 2 and 50. No q_{peak} was observed for gels with Mn^{2+} and Zn^{2+} at $n_{Cl}/n_M = 50$, indicating nanocrystals remained dispersed at room temperature. For the remaining samples, the average characteristic length is 12.0 ± 0.2 nm.

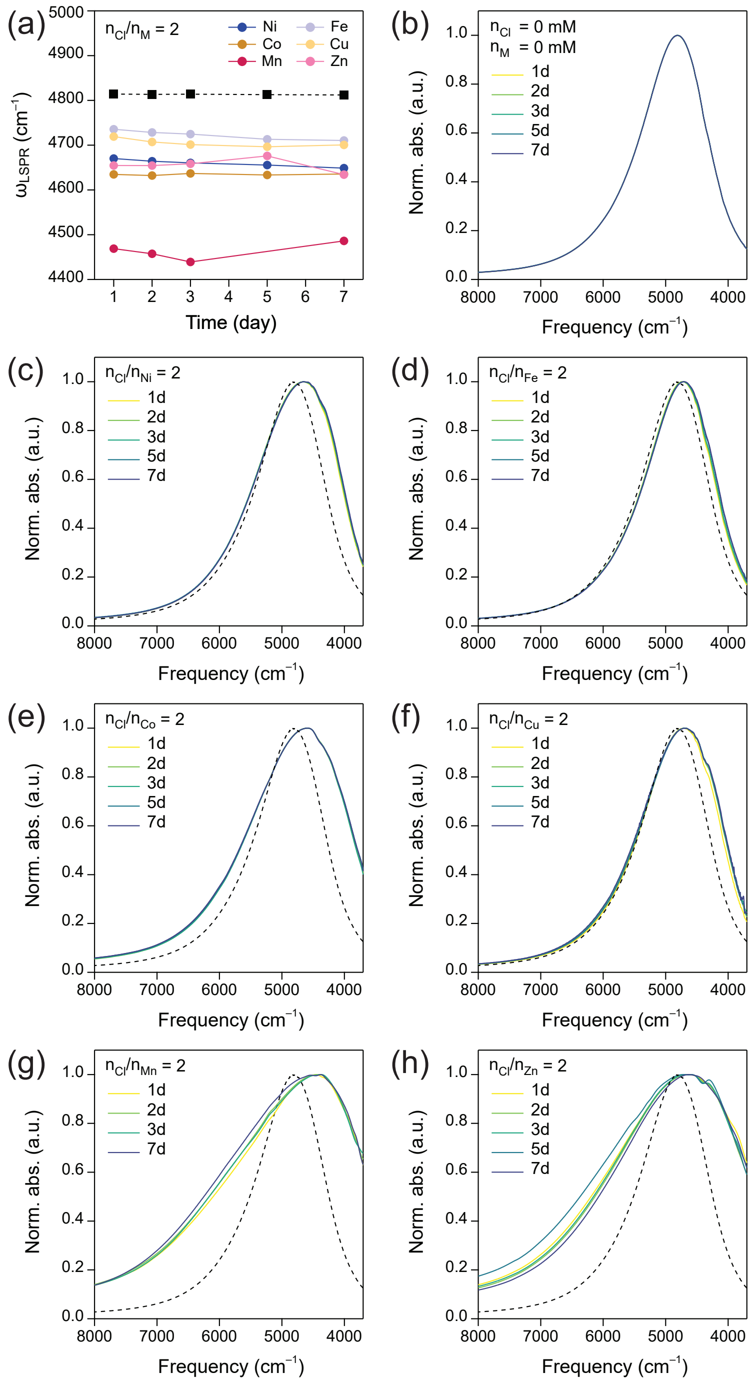

Figure S11: **Infrared response of TL-ITO at a chloride-to-metal ion molar ratio of 2.** (a) Time-dependent LSPR peak frequency (ω_{LSPR}) of TL-ITO following the addition of various metal ions. TL-ITO without metal ion addition is shown as black squares for reference. (b-h) Infrared response of TL-ITO with (b) no added metal ion, (c) Ni^{+2}, (d) Fe^{+2}, (e) Co^{+2}, (f) Cu^{+2}, (g) Mn^{+2}, and (h) Zn^{+2}. For comparison, the spectrum of TL-ITO without metal ion addition on day 7 is shown as dashed curves in panels (c–h).

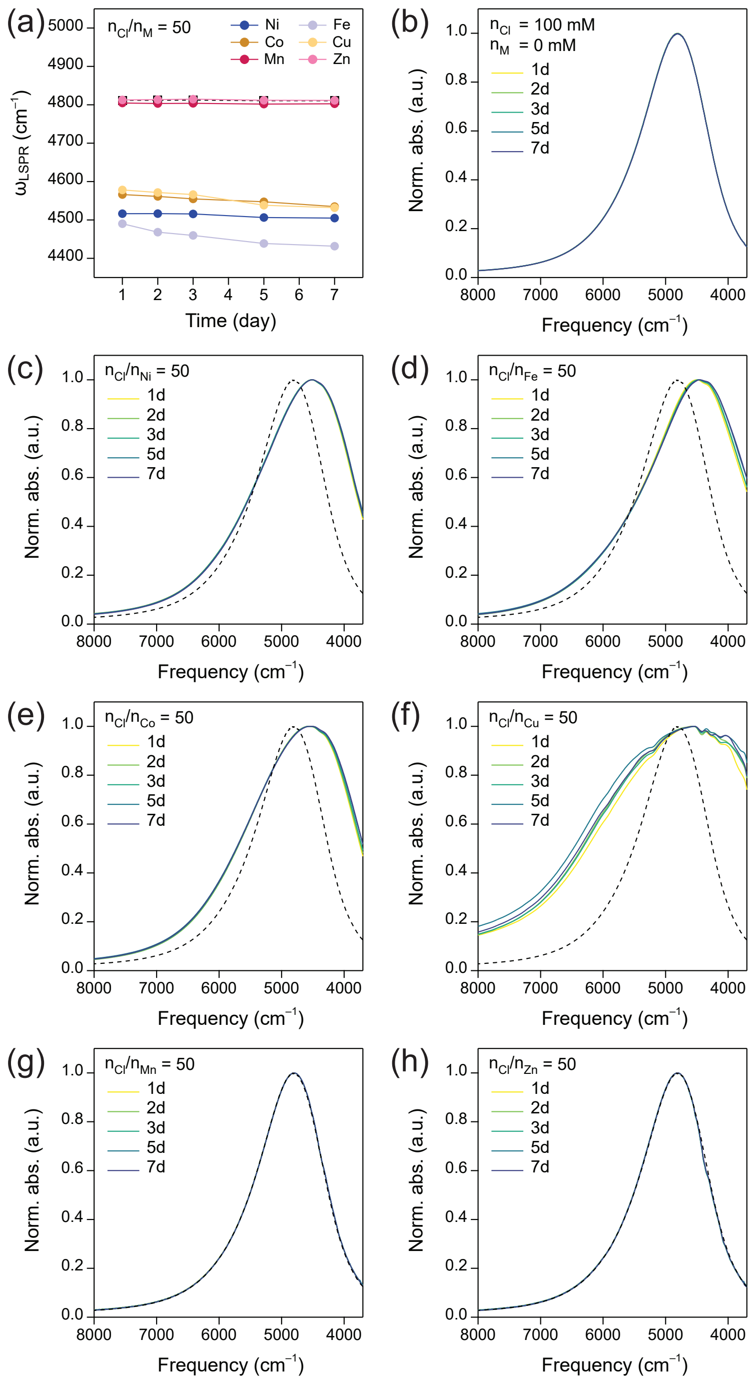

Figure S12: **Infrared response of TL-ITO at a chloride-to-metal ion molar ratio of 50.** (a) Time-dependent LSPR peak frequency (ω_{LSPR}) of TL-ITO following the addition of various metal ions. TL-ITO without metal ion addition is shown as black squares for reference. (b-h) Infrared response of TL-ITO with (b) no added metal ion, (c) Ni^{+2}, (d) Fe^{+2}, (e) Co^{+2}, (f) Cu^{+2}, (g) Mn^{+2}, and (h) Zn^{+2}. For comparison, the spectrum of TL-ITO without metal ion addition on day 7 is shown as dashed curves in panels (c–h).

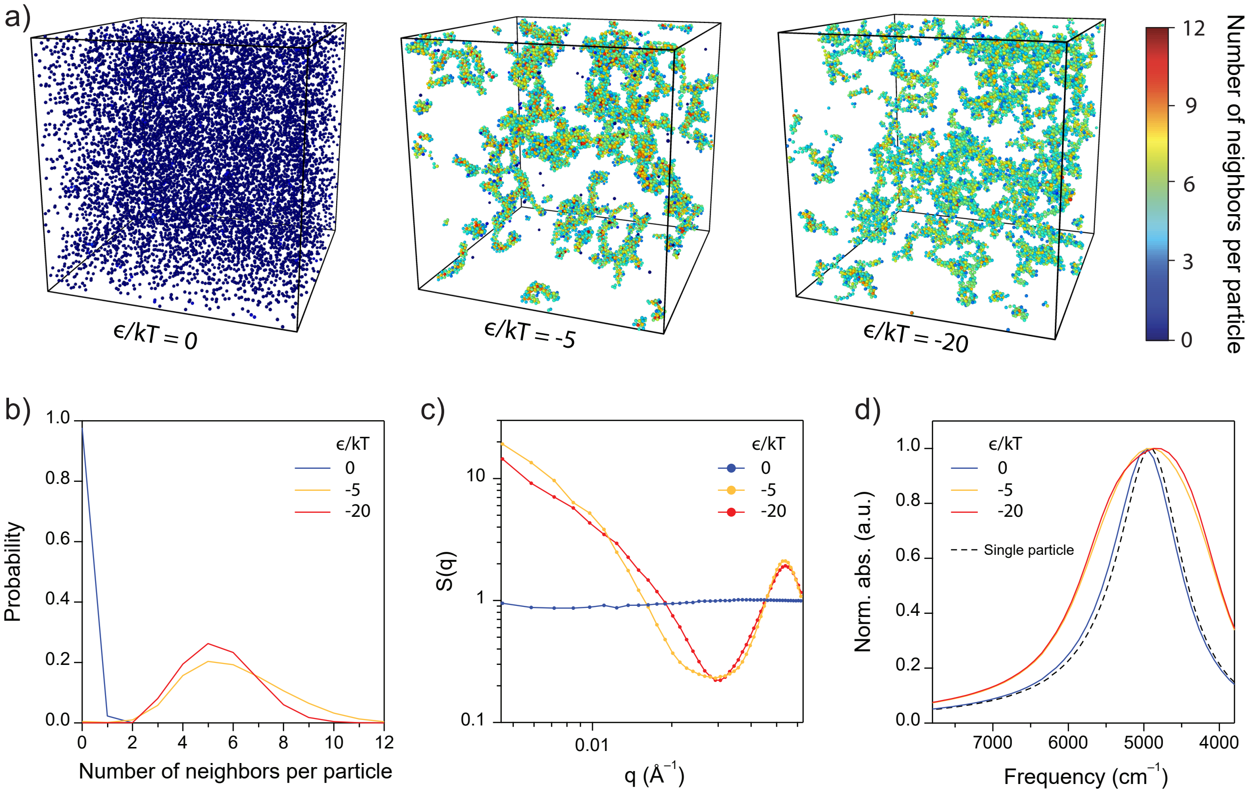

Figure S13: **Local gel structure and optical evolution at different interparticle attraction strengths** (a) Snapshots from kMC simulations showing nanocrystal assembly with interparticle attraction strengths $\epsilon = 0kT$ (left), $\epsilon = -5kT$ (middle), and $\epsilon = -20kT$ (right). The crawling time is fixed at $\tau_{\mathrm{crawl}}/\tau_0 = 1$. The color scale indicates the number of neighbors per particle. The structure evolves from dispersion ($\epsilon = 0kT$) to gels ($\epsilon = -5kT$ and $\epsilon = -20kT$), while denser gel structure is observed at intermediate interaction strengths ($\epsilon = -5kT$). (b) The number of neighbors per particle with different interparticle attraction strengths. (c) Static SAXS $S(q)$ calculated from kMC simulation at the onset of percolated clusters (except for $\epsilon = 0kT$, where the nanoparticles stay in the dispersion state). e) LSPR extinction spectra of the structures calculated using the mutual polarization method. Single particle spectrum (dashed) is shown for reference.

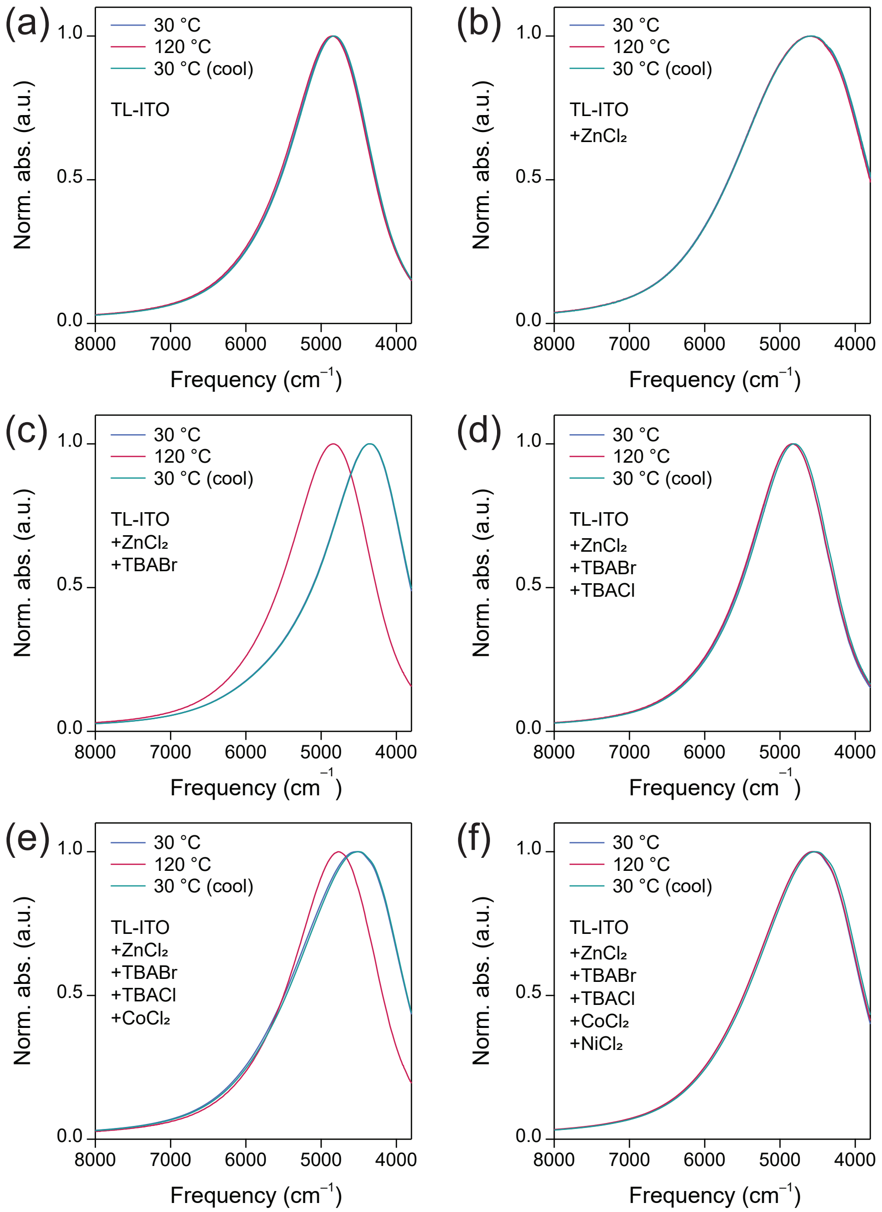

Figure S14: **Infrared response of TL-ITO upon sequential addition of different metal and halide ions.** LSPR absorption spectra of TL–ITO measured at 30 °C (blue), 120 °C (red), and again at 30 °C (green) for (a) a control sample without added salts, and after cumulative addition of (b) $ZnCl_2$, (c) TBABr, (d) TBACl, (e) $CoCl_2$, and (f) $NiCl_2$. Final concentrations were: TL-ITO (2 nM), $ZnCl_2$ (2 mM), TBABr (10 mM), TBACl (100 mM), $CoCl_2$ (2 mM), and $NiCl_2$ (2 mM). All spectra were recorded after 5 minutes of thermal equilibration at the target temperature.

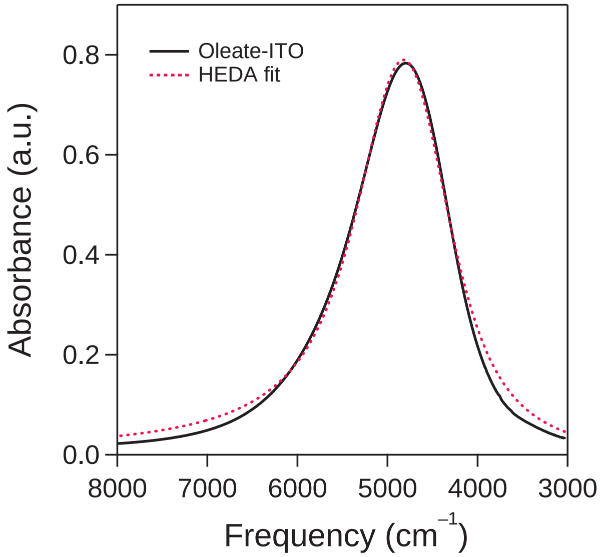

Figure S15: **A dielectric model fit to LSPR absorption spectrum of oleate-ITO dispersed in tetrachloroethane.** Infrared absorption spectra of oleate-ITO dispersed in tetrachloroethylene (solid) and its fit to a core-shell Drude dielectric model, HEDA, heterogeneous ensemble Drude approximation (dashed).[S13] Fit results can be found in Table S1.

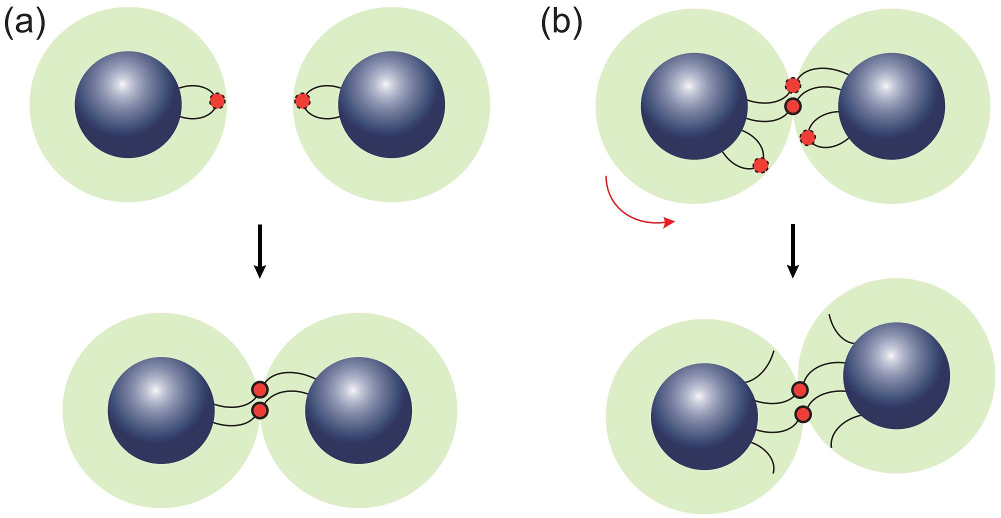

Figure S16: **Association and crawling upon assembly** (a) Interparticle association is where two nanoparticles collide and form interparticle bridges. This requires two self loops (one on each particle) to first break. (b) Crawling is where nanoparticles rearrange their relative positions while remaining "in contact." The elementary step requires breaking one bridge and two self loops (one on each particle) to form a new bridge.

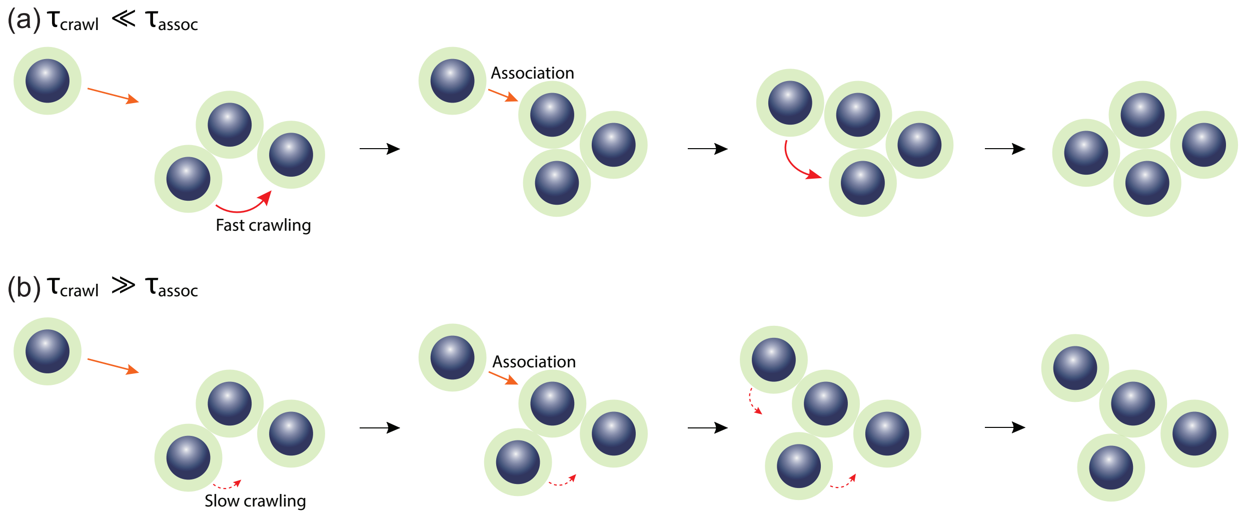

Figure S17: **Competition between crawling and association.** Structural evolution when a single particle is added to a cluster of three particles for two cases: (a) $\tau_{\text{crawl}} \ll \tau_{\text{assoc}}$ and (b) $\tau_{\text{crawl}} \gg \tau_{\text{assoc}}$. Fast crawling in (a) alters the internal structure of the cluster as it minimizes its energy, leading to a denser configuration than in (b).

Table S1: **Dielectric parameters of nanocrystals from a HEDA fit.** d_{core}, nanocrystal inorganic core diameter; ω_p, plasma frequency; γ, damping constant; η_c, electron accessible volume fraction of nanocrystal cores inside surface depletion region. Sn doping concentration and d_{core}, input parameters for HEDA fitting, were determined by ICP-OES and SAXS, respectively.

Sn doping (at.%)	d_{core} (nm)	ω_p (cm^{-1})	γ (cm^{-1})	η_c
4.8	12.1 ± 1.2	14181	1066	0.912

References

(1) Jansons, A. W.; Hutchison, J. E. Continuous growth of metal oxide nanocrystals: enhanced control of nanocrystal size and radial dopant distribution. *ACS Nano* **2016**, *10*, 6942–6951.

(2) Dominguez, M. N.; Howard, M. P.; Maier, J. M.; Valenzuela, S.; Sherman, Z. M.; Reimnitz, L. C.; Kang, J.; Cho, S. H.; Gibbs, S. L.; Menta, A. K.; Zhuang, D. L.; van der Stok, A.; Kline, S. J.; Anslyn, E. V.; Truskett, T. M.; Milliron, D. J. Assembly of linked nanocrystal colloids by reversible covalent bonds. *Chem. Mater.* **2020**, *32*, 10235–10245.

(3) Kang, J.; Valenzuela, S. A.; Lin, E. Y.; Dominguez, M. N.; Sherman, Z. M.; Truskett, T. M.; Anslyn, E. V.; Milliron, D. J. Colorimetric quantification of linking in thermoreversible nanocrystal gel assemblies. *Sci. Adv.* **2022**, *8*, eabm7364.

(4) Kang, J.; Sherman, Z. M.; Crory, H. S.; Conrad, D. L.; Berry, M. W.; Roman, B. J.; Anslyn, E. V.; Truskett, T. M.; Milliron, D. J. Modular mixing in plasmonic metal oxide nanocrystal gels with thermoreversible links. *J. Chem. Phys.* **2023**, *158*, 024903.

(5) Kang, J.; Sherman, Z. M.; Conrad, D. L.; Crory, H. S.; Dominguez, M. N.; Valenzuela, S. A.; Anslyn, E. V.; Truskett, T. M.; Milliron, D. J. Structural control of plasmon resonance in molecularly linked metal oxide nanocrystal gel assemblies. *ACS Nano* **2023**, *17*, 24218–24226.

(6) Chandrasekhar, S. Stochastic Problems in Physics and Astronomy. *Reviews of Modern Physics* **1943**, *15*, 1–89.

(7) Jha, P. K.; Kuzovkov, V.; Grzybowski, B. A.; Olvera De La Cruz, M. Dynamic self-assembly of photo-switchable nanoparticles. *Soft Matter* **2012**, *8*, 227–234.

(8) Sciortino, F.; Zhang, Y.; Gang, O.; Kumar, S. K. Combinatorial-entropy-driven aggregation in DNA-grafted nanoparticles. *ACS Nano* **2020**, *14*, 5628–5635.

(9) Sherman, Z. M.; Kim, K.; Kang, J.; Roman, B. J.; Crory, H. S.; Conrad, D. L.; Valenzuela, S. A.; Lin, E.; Dominguez, M. N.; Gibbs, S. L.; Anslyn, E. V.; Milliron, D. J.; Truskett, T. M. Plasmonic response of complex nanoparticle assemblies. *Nano Lett.* **2023**, *23*, 3030–3037.

(10) Sherman, Z. M.; Milliron, D. J.; Truskett, T. M. Distribution of single-particle resonances determines the plasmonic response of disordered nanoparticle ensembles. *ACS Nano* **2024**, *18*, 21347–21363.

(11) Gibbs, S. L.; Staller, C. M.; Agrawal, A.; Johns, R. W.; Saez Cabezas, C. A.; Milliron, D. J. Intrinsic optical and electronic properties from quantitative analysis of plasmonic semiconductor nanocrystal ensemble optical extinction. *J. Phys. Chem. C* **2020**, *124*, 24351–24360.

(12) De Roo, J.; Yazdani, N.; Drijvers, E.; Lauria, A.; Maes, J.; Owen, J. S.; Van Driessche, I.; Niederberger, M.; Wood, V.; Martins, J. C.; Infante, I.; Hens, Z. Probing solvent–ligand interactions in colloidal nanocrystals by the NMR line broadening. *Chem. Mater.* **2018**, *30*, 5485–5492.

(13) Gibbs, S. L.; Staller, C. M.; Agrawal, A.; Johns, R. W.; Saez Cabezas, C. A.; Milliron, D. J. Intrinsic optical and electronic properties from quantitative analysis of plasmonic semiconductor nanocrystal ensemble optical extinction. *J. Phys. Chem. C* **2020**, *124*, 24351–24360.
